# Foot-and-Mouth Disease Virus Persists in the Light Zone of Germinal Centres

**DOI:** 10.1371/journal.pone.0003434

**Published:** 2008-10-20

**Authors:** Nicholas Juleff, Miriam Windsor, Elizabeth Reid, Julian Seago, Zhidong Zhang, Paul Monaghan, Ivan W. Morrison, Bryan Charleston

**Affiliations:** 1 Pirbright Laboratory, Institute for Animal Health, Woking, Surrey, United Kingdom; 2 Centre for Tropical Veterinary Medicine, University of Edinburgh, Easter Bush Veterinary Centre, Roslin, Midlothian, United Kingdom; Institut Pasteur Korea, Republic of Korea

## Abstract

Foot-and-mouth disease virus (FMDV) is one of the most contagious viruses of animals and is recognised as the most important constraint to international trade in animals and animal products. Two fundamental problems remain to be understood before more effective control measures can be put in place. These problems are the FMDV “carrier state” and the short duration of immunity after vaccination which contrasts with prolonged immunity after natural infection. Here we show by laser capture microdissection in combination with quantitative real-time reverse transcription polymerase chain reaction, immunohistochemical analysis and corroborate by *in situ* hybridization that FMDV locates rapidly to, and is maintained in, the light zone of germinal centres following primary infection of naïve cattle. We propose that maintenance of non-replicating FMDV in these sites represents a source of persisting infectious virus and also contributes to the generation of long-lasting antibody responses against neutralising epitopes of the virus.

## Introduction

Foot-and-mouth disease virus (FMDV) causes an acute vesicular disease which is endemic throughout large parts of Asia, Africa and South America [1]. Although the disease has been eradicated in Europe and North America, introduction of infection, as occurred in the United Kingdom in 2001, can cause devastating outbreaks of disease [2]. Despite the availability of vaccines, control of such outbreaks continues to rely on detection and slaughter of affected herds [1]. One of the features of FMDV infection that has a major impact on control policies is the “carrier state” [3]. A carrier of FMDV is defined as an animal from which live-virus can be recovered from scrapings of the oropharynx after 28 days following infection [4]. Over 50% of ruminants exposed to viral challenge, whether vaccinated or not, can become carriers [1]. Recovery of infectious virus from oropharyngeal scrapings of foot-and-mouth disease (FMD) recovered cattle is intermittent and the titre of virus recovered from carrier animals is low, often falling below the level thought to be necessary for successful transmission to susceptible animals [5]. Intermittent virus recovery may be related to the heterogeneous nature of oropharyngeal samples with saliva, mucus and cells present in varying quantities [3]. Although such carrier animals have never convincingly been directly demonstrated to transmit infection, they are perceived as a potential source of new infections and consequently there is a reluctance to use vaccination as a primary means of controlling outbreaks in disease-free countries [3]. Despite the potential epidemiological and immunological significance, very little is known about the mechanism by which the “carrier state” is established or maintained. In one series of experiments, carriers were treated with dexamethasone in order to depress their immune systems, and kept in contact with susceptible cattle, but this had the reverse effect of causing the virus to disappear from oropharyngeal scrapings, only to reappear once the treatment was stopped [6]. There was no transmission between carrier and susceptible cattle. Despite the uncertainty concerning the capacity of the carrier animals to transmit virus, there is a requirement to identify and remove these animals before a country or region can declare freedom from infection and resume international animal trade. Hence the infection status of a country can have a profound impact on its economy [2].

Virus is cleared rapidly from blood during the acute stage of FMD, coinciding closely with the emergence of an antiviral antibody response. Viral RNA is detected in the blood of infected cattle, using real-time reverse transcription polymerase chain reaction (rRT-PCR), but becomes undetectable from as early as 3 to 5 days after onset of clinical signs. This is in contrast to pharyngeal tissue including the soft palate, nasopharynx, oropharynx, palatine tonsil and mandibular lymph node which have been shown to contain viral RNA for up to 72 days after infection [7]. The significance of continued detection of viral RNA has not been clear since FMDV proteins have not been detected, in previous studies in these tissues, following the resolution of vesicular lesions. Importantly, prior to this publication, FMDV proteins have not been detected previously in lymphoid tissue *in vivo* at any stage of infection and viral proteins have not been detected in any tissue following resolution of vesicular lesions.

## Results

### Laser capture microdissection

**Figure 1:**
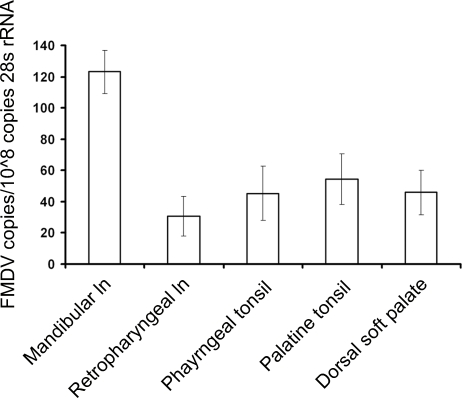
Analysis of tissue 38 days post contact infection by LCM in combination with quantitative rRT-PCR. FMDV copies per 10^8^ copies of 28 s rRNA of GC samples positive for FMDV by quantitative rRT-PCR versus tissue type from cattle 38 days post contact infection (n = 4 animals. Adjusted means±standard error of the mean; ANOVA, general linear model). Approximately 100 bovine peripheral blood mononuclear cells contain 10^8^ copies of 28 s rRNA. There was a statistically significant association between the quantity of FMDV genome present in GC samples and type of tissue (p = 0.0039, Fisher's exact test).

Germinal centre (GC) and non-GC regions of the dorsal surface of the *palatum molle* (dorsal soft palates), pharyngeal tonsils [8], palatine tonsils, lateral retropharyngeal lymph nodes and mandibular lymph nodes obtained from four cattle 38 days post contact exposure to FMDV serotype O were selected for laser capture microdissection (LCM, Table 1, Figure S1). FMDV genome was detected consistently by quantitative rRT-PCR within the GC samples obtained by LCM (Figure S2 to S6). No FMDV genome was detected in the epithelium of the dorsal soft palates and pharyngeal tonsils (Figure S2 to S3). No FMDV genome was detected in the crypt epithelium, glandular epithelium and interfollicular regions of the palatine tonsils or the interfollicular regions of the mandibular lymph nodes and lateral retropharyngeal lymph nodes (Figure S4 to S6). No FMDV genome could be detected in GC samples obtained by LCM from non-infected control animals (data not shown). Significantly more FMDV genome copies per 10^8^ copies of 28 s rRNA were detected in replicates of six GCs from mandibular lymph nodes, compared to similar replicates harvested from other tissue (Figure 1) (Mandibular lymph node compared to lateral retropharyngeal lymph node [p = 0.0014], mandibular lymph node compared to palatine tonsil [p = 0.0376], mandibular lymph node compared to pharyngeal tonsil [p = 0.0392] and mandibular lymph node compared to dorsal soft palate [p = 0.0148]; ANOVA, Tukey simultaneous test).

**Table 1:** Laser microdissected GC samples processed by quantitative rRT-PCR to detect FMDV*.

Tissue	Number of positive replicates	Number of negative replicates	Threshold cycle values of positive replicates
DSP	9	3	38.74 to 46.24
Pharyngeal tonsils	6	6	36.76 to 40.22
Palatine tonsils	7	5	35.73 to 39.92
RPLN	12	0	34.68 to 37.01
MLN	12	0	35.64 to 40.03

* Measured at 38 days post contact infection (n = 4 animals). Only GC samples were found to contain FMDV genome after 50 cycles [33].

DSP = dorsal soft palate.

RPLN = lateral retropharyngeal lymph node.

MLN = mandibular lymph node.

### *In situ* hybridization

**Figure 2:**
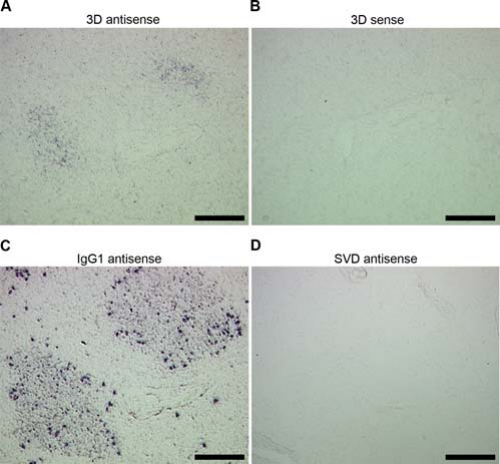
Tissue localisation of FMDV by *in situ* hybridization. Consecutive mandibular lymph node frozen sections harvested from cattle 38 days post contact infection. (A) 3D antisense RNA probe detecting sense FMDV 3D RNA (see Figure S15 for high power images). (B) Lack of staining following hybridization with 3D sense RNA control probe. (C) Positive staining of IgG1 mRNA in GC B-cells after hybridization with IgG1 antisense RNA positive control probe. (D) Lack of staining following hybridization with SVD antisense control probe. No counterstain. Scale bars, (A, B, C) = 200 µm, (D) = 500 µm.

An optimised detection protocol with tyramide signal amplification was compared to conventional chromagenic detection (Figure S7) [9]. Tyramide signal amplification combined with endogenous peroxidase and alkaline phosphatase quenching enhanced the specific hybridization signal and reduced background signal compared to conventional detection. The FMDV 3D antisense RNA probes, control 3D sense probes and control SVD RNA probes [9] were validated on FMDV infected and mock infected BHK-21 cells and on tissue samples collected from non-infected and FMDV infected animals (Figure S8 and S9). Despite the obvious signal obtained when detecting positive strand viral RNA in infected cells, it was difficult to detect negative strand viral RNA by *in situ* hybridization. *In situ* hybridization of dorsal soft palates, mandibular lymph nodes, palatine tonsils, pharyngeal tonsils and lateral retropharyngeal lymph nodes from ten animals 14 to 38 days post contact infection supported the LCM results. FMDV 3D RNA was identified in GCs of mandibular lymph node, palatine tonsil and lateral retropharyngeal lymph node sections but not in other compartments of these tissues (Figure 2).

### Immunofluorescence confocal microscopy

To determine whether viral RNA was associated with viral structural proteins; frozen sections from the dorsal soft palates, pharyngeal tonsils, palatine tonsils, lateral retropharyngeal lymph nodes and mandibular lymph nodes collected 29 to 38 days post contact infection were analysed using a new set of virus-specific monoclonal antibodies (MAbs) shown to be specific for conformational, non-neutralising epitopes of the FMDV capsid. These new MAbs immunoprecipitated FMDV capsid proteins, consistent with previously published antibodies (Figure S10), yet did not detect FMDV proteins by western blot and were non-neutralising (data not shown). Also, the MAbs readily detected virus in bovine tongue during acute infection (Figure S11), virus infected cells (Figure S12) and rare infected cells in lymphoid tissue during acute infection (Figure S13). The ability of the MAbs specific for FMDV capsid to detect immune complexed virus on the surface of cells in culture was also confirmed (Figure S14).

**Figure 3:**
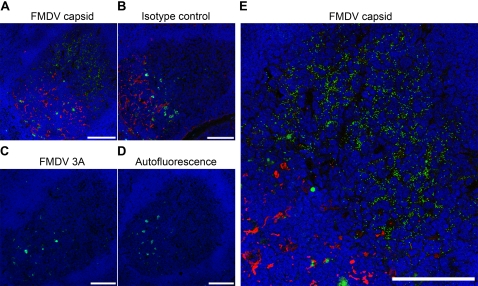
Tissue localisation of FMDV proteins 29–38 days post infection. Mandibular lymph node GC sections 38 days post contact infection. (A) FMDV capsid labelled green with MAb IB11, dark zone FDCs labelled red with MAb D46 [13]. (B) dark zone FDCs labelled red with MAb D46, no signal detected with isotype primary control MAb TRT1 labelled green [38]. (C) No signal detected with FMDV non-structural protein 3A labelled green with MAb 2C2 [10]. FMDV non-structural proteins could not be detected by immunohistochemical analysis of tissue from 29 to 38 days post contact infection. (D) No primary or secondary antibodies highlighting autofluorescence associated with bovine GCs. (E) High power image of (A). Nuclei stained blue (DAPI), scale bars = 100 µm.

**Figure 4:**
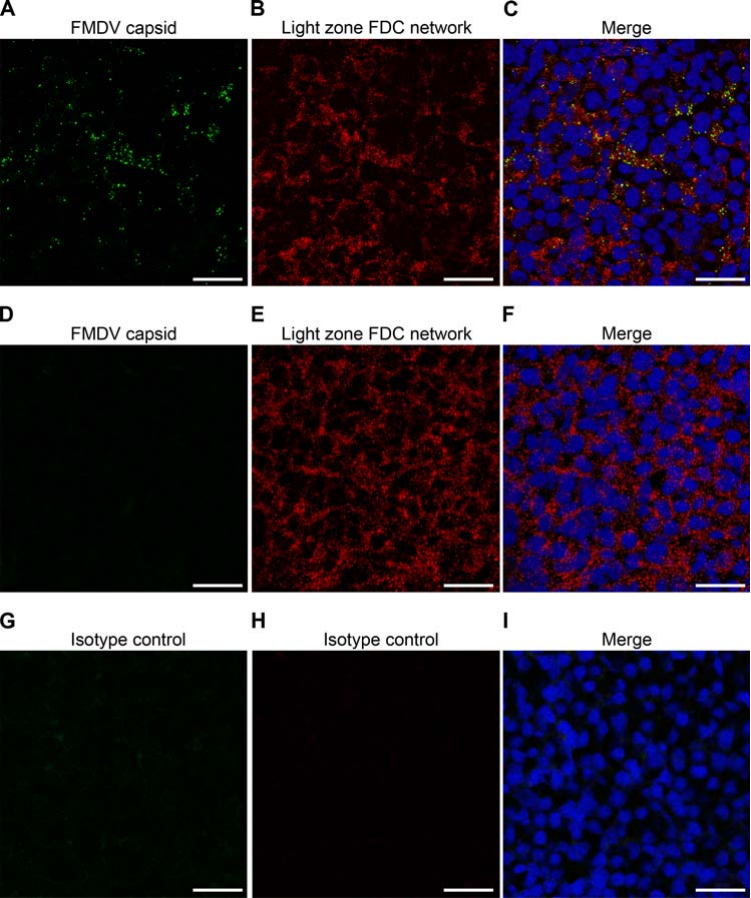
Localisation of FMDV capsid proteins within the light zone FDC network 29–38 days post infection. (A–C) Mandibular lymph node frozen sections 38 days post infection. (A) FMDV capsid protein labelled green with MAb IB11. (B) Light zone FDC network labelled red with MAb CNA.42 [13]. (C) Merge image showing the diffuse punctate pattern associated with FMDV capsid (green) linked to the light zone FDC network (red). (D–F) Non-infected control tissue. (D) No signal detected using MAb IB11 on control tissue. (G–I) No signal was detected on infected mandibular lymph node sections with isotype matched control MAbs TRT1 (G) [38], or AV48 (H). Nuclei stained blue (DAPI), scale bars = 20 µm.

The anti-FMDV capsid MAbs gave a diffuse punctate pattern of positive labelling which was restricted to GCs within lymphoid tissue and confined to the light zone within the GC from 29 days post infection (Table 2, Figure 3, 4 and S15). By contrast, the FMDV non-structural proteins 3A and 3C could not be detected in any of the tissues from animals after 28 days post contact infection (Table 2, Figure 3) [10], [11]. Antibodies specific for 3A and 3C readily detected infected cells in FMDV vesicles and lymph tissue during the acute phase of infection and in infected BHK-21 cells, co-localising with FMDV capsid (Figure S11 to S13). Also, using immunofluorescence confocal microscopy there was a lack of labelling for the dominant FMDV cellular receptor, αvβ6 integrin [12], in the GCs (Figure S16). The diffuse punctate pattern of labelled viral capsid was shown to be localised to the light zone follicular dendritic cell (FDC) network by co-labelling with an antibody specific for light zone FDCs (Figure 4) [13]. Detailed analysis of *in situ* hybridization and immunohistochemistry showed a consistent punctate pattern (Figure S15). The punctate labelling pattern observed in Figure S15 panel D is consistent with the distribution pattern of iccosomes on FDCs [14]. This pattern is in contrast to the diffuse cytoplasmic labelling pattern of cells observed during acute infection *in vivo* and in infected cells *in vitro* (Compare Figures 3 and 4 with Figure S11 to S13).

**Table 2:** Immunohistochemical analysis of tissue 29 to 38 days post contact infection for FMDV capsid and non-structural proteins*.

Tissue	Number of animals sampled	FMDV capsid+ve GCs**
DSP	17	0
Pharyngeal tonsils	10	0
Palatine tonsils	10	6
RPLN	10	8
MLN	22	22

* Tissue was negative by immunohistochemical analysis for FMDV non-structural proteins.

** Number of animals with GCs positive for FMDV capsid.

DSP = dorsal soft palate.

RPLN = lateral retropharyngeal lymph node.

MLN = mandibular lymph node.

## Discussion

We have shown that FMDV genome, using LCM and quantitative rRT-PCR, can be detected consistently in GCs within the dorsal soft palate, pharyngeal tonsil, palatine tonsil, lateral retropharyngeal lymph node and mandibular lymph node at 38 days post contact infection. Also, FMDV genome in these tissues was restricted to the GC. These findings were confirmed with *in situ* hybridization studies, which revealed FMDV 3D RNA in GCs of lymphoid tissues but not in other compartments of these tissues. Using MAbs specific for conformational, non-neutralising epitopes of the FMDV capsid, we identified viral structural proteins restricted to the light zone FDC network of GCs within mandibular lymph nodes, lateral retropharyngeal lymph nodes and palatine tonsils up to 38 days post contact infection, but not in the dorsal soft palates or pharyngeal tonsils. The inability to detect FMDV capsid in the dorsal soft palates and pharyngeal tonsils by immunohistochemistry is in contrast to the clear detection of FMDV genome by LCM. This inconsistency may be a consequence of differences in assay sensitivity or genomic RNA persisting longer than virus [15]. The diffuse punctate pattern of labelled viral capsid in tissue from 29 to 38 days post infection, similar to the FMDV genome staining pattern detected by *in situ* hybridization, was in contrast to the diffuse cytoplasmic pattern observed in cells during acute infection *in vivo* and in infected cells *in vitro*.

The mandibular lymph nodes had notably more GCs containing FMDV capsid compared to the lateral retropharyngeal lymph nodes and palatine tonsils. This is consistent with the detection of significantly more FMDV genome copies/10^8^ copies of 28 s rRNA in replicates of six GCs from mandibular lymph nodes, compared to similar replicates harvested from other tissues. FMDV capsid was detected in mandibular lymph node GCs of all animals examined between 29 to 38 days post contact infection (n = 22), including five animals where FMDV could not be recovered by virus isolation or detected by rRT-PCR analysis of oropharyngeal scrapings collected at post-mortem 29 to 34 days post infection using probang sampling cups [3]. These results indicate that virus is likely to persist in all cattle to some degree following infection. This predilection to the mandibular lymph node is not surprising because afferent lymphatics of the mandibular lymph nodes in the bovine drain the oral cavity and tongue, which are important sites of viral replication during the acute phase of infection. However, these results do not support findings from previous studies which reported detection of viral RNA by *in situ* hybridization and whole tissue quantitative rRT-PCR in the dorsal soft palate epithelium in carrier animals [7]. We did not detect viral RNA in the epithelial compartments of all the tissues examined either by LCM and quantitative rRT-PCR or *in situ* hybridization, although we routinely detected viral RNA and capsid in GCs of these tissues.

Although MAbs specific for FMDV non-structural proteins 3A and 3C could detect infected cells *in vitro* and *in vivo* during the acute phase of infection, no FMDV non-structural proteins were detected in any of the tissues examined from 29 days post contact infection. The absence of detectable FMDV non-structural proteins indicates that the presence of viral RNA is not associated with active viral replication [10], [11]. The finding of close co-localisation of viral RNA and capsid conformational epitopes, in the absence of non-structural proteins, supports the hypothesis that FMD viral particles or immune complexes are maintained in GC light zones in a non-replicating state. Interestingly, FMDV capsid was detected in the light zone of mandibular lymph node GCs as early as 3 to 4 days post intradermolingual challenge (n = 4).

FMDV is known to use members of the integrin family to initiate infection [12]. Current evidence from *in vitro* and *in vivo* studies indicates that αvβ6 integrin serves as the major cellular receptor for FMDV. Since the distribution of αvβ6 expression in cattle, namely in epithelial cells in the tongue, interdigital skin and coronary band [12] correlates closely with the sites of FMDV replication, it is thought to determine the tissue tropism of the virus. We have shown by immunofluorescence confocal microscopy that αvβ6 is not expressed in GCs, indicating that the early localisation of FMDV to GC light zones is independent of αvβ6 expression. Binding of virus to light zone GC cells during the early stages of infection may play an important role in facilitating a FMDV B-cell response [16], [17].

The results of these studies have important implications for understanding both the mechanism of viral persistence and the ability of FMDV infection to stimulate long-lasting antibody responses. FDCs are known to be non-endocytic cells capable of capturing and retaining antigen in the form of immune complexes for long periods of time [18], [19]. Retention of immune complexed FMDV particles within lymphoid tissue represents a possible source of the infectious material detected by pharyngeal sampling of infected cattle either by direct harvesting of mucosal associated lymphoid tissue GCs or sampling of secondary cells, for example macrophages, dendritic cells or B-cells, able to support a low level virus replication cycle in the presence of high levels of neutralising antibodies [20], [21]. These cell types may also act as a source of infectious material capable of seeding other remote sites for ongoing low level viral replication in the pharynx. Of the tissue examined in the present study, only material from the palatine tonsils and pharyngeal tonsils are likely to be represented in probang samples. Viral RNA was detected in GCs of palatine tonsils and pharyngeal tonsils but capsid antigen was only detected in GCs of palatine tonsils making this tissue a likely source of infectious virus detected by probang sampling in cattle. FDCs are notoriously difficult cells to work with, so far we have been unable to rescue infectious or immune complexed FMDV from lymphoid tissue most likely due to technical difficulties working with the bovine system. Retention of other viruses such as HIV in a replication-competent state within the light zone of GCs has been reported and the next step will require the development and interrogation of murine model systems [22]. The previous observation that dexamethasone treatment suppresses the ability to detect FMDV in oropharyngeal scrapings [6] is consistent with the hypothesis that the GC is the reservoir for infectious virus, since glucocorticoid administration to mice is known to result in atrophy of the FDC network [23]. The recrudescence of virus in pharyngeal scrapings after dexamethasone treatment could be a consequence of the failure of the treatment to completely eliminate structures capable of maintaining viable virus. FDC-trapped HIV has been shown to represent a significant reservoir of infectious and highly diverse HIV, demonstrating greater genetic diversity than most other tissues, providing drug-resistant and immune-escape quasispecies that contribute to virus transmission, persistence and diversification [24]. Retention of intact FMDV particles on the FDC network would therefore provide an ideal mechanism of maintaining a highly cytopathic and lytic virus like FMDV extracellularly in a non-replicating, native, stable nondegraded state [22], [25]. This reservoir could serve as a source of genetically diverse viral mutants (quasispecies) able to infect susceptible cells that come into contact with the FDC network [26], [27].

FMDV infection in ruminants elicits an immune response that can provide protection for several years [28] and the level of protection correlates well with specific serum neutralising antibody titres (SNTs) [1]. This is in contrast to vaccination, with current FMDV vaccines prepared with inactivated virus and adjuvants, providing short term duration of SNTs and protection [29]. Long-term maintenance of elevated, specific antibody levels in mice following acute vesicular stomatitis virus (VSV) infection has been shown to be associated with the co-localisation of antigen with specific memory B-cells within long-lived GCs [30]. VSV is a cytolytic virus that does not persist in an infectious form in mice, thus highlighting the function of FDC trapping and retention serving as a long-term repository of immunogenic antigen for maintenance of SNTs. Hence, efficient retention within the GCs of intact viral capsids, as opposed to the constituent viral proteins, may be a requirement for sustaining antibody responses relevant for providing protection against challenge. Indeed, in a recent review of the functional significance of antigen retained on FDC, Kosco-Vilbois suggests the observation that B-cell responses are independent of FDC-associated antigen is only valid in mice that are immunised with forms of antigen that leave persistent depots [31]. Therefore, we believe that long-term antibody responses detectable after FMDV infection are maintained in part by antigen persisting on FDCs. Based on the evidence presented here we suggest the persistence of FMDV after acute infection is both a consequence of the host immune response and a requirement for the long-term maintenance of protective virus-specific antibody responses.

## Materials and Methods

### Challenge

The cattle examined in this study were infected by close contact with cattle infected with FMDV isolates O/UKG/34/2001 or O1BFS1860. Animal experimentation was approved by the Institute for Animal Health (IAH) ethical review board under the authority of a Home Office project licence in accordance to the Home Office Guidance on the Operation of the Animals (Scientific Procedures) Act 1986 and associated guidelines.

### Laser capture microdissection

LCM was performed as described previously [32], except that ethanol fixed frozen sections were stained with a 1% solution of toluidine blue (Fluka) for 3 minutes, washed for 30 seconds in nuclease free water, dehydrated by a graded ethanol series and allowed to air dry for 10 minutes. Three replicates of the different tissues regions, each containing six microdissected samples collected in the caps of RNase-free PCR tubes (Ambion), were collected from each tissue for RNA isolation with the RNeasy Micro Kit (Qiagen) and processed by quantitative rRT-PCR.

### Quantitative rRT-PCR

Reverse transcription of isolated RNA was performed concurrently with 10-fold dilution series of FMDV and bovine 28 s standard RNA (Applied Biosystems). Standard RNA of FMDV O/UKG/34/2001 internal ribosomal entry site was prepared as previously described [33]. Bovine 28 s standard RNA was synthesised from a plasmid containing a 261 base pair PCR product insert of bovine peripheral blood mononuclear cell 28 s ribosomal RNA. Linearised plasmid was transcribed with a Megascript® T7 kit (Ambion) and subjected to TURBO DNase™ treatment and removal reagents (Ambion). The molecular weight of the entire 295 nt product was calculated and number of copies/ml determined as previously described [34]. FMDV primer/probe sets and detection were as previously described; samples with no detectable fluorescence above threshold after 50 cycles were taken to be negative [33]. 28 s primer/probe sets and detection were adapted from methods previously described [35]. There was no statistically significant association between FMDV genome copies expressed as FMDV copies/10^8^ copies of 28 s rRNA and amount of 28 s rRNA per reaction (p = 0.206. ANOVA, general linear model).

### Selection of MAbs specific for conformational, non-neutralising epitopes of the FMDV capsid

Mouse MAbs IB11, FC6, AD10 and BF8 raised against 146 S FMDV type O1 antigen were selected by ELISA and virus neutralising antibody test as described in the Office International des Epizooties (OIE) Manual of Diagnostic Tests and Vaccines for Terrestrial Animals, 5^th^ edition, 2004. Immunoprecipitation analysis was performed as previously described by Rouiller *et al* [36], BHK-21 cells were infected with O1BFS at MOI 5 for four hours in total and pulsed with 35S methionine/cysteine for two of these hours. Cells were lysed and immunoprecipitated with D9 (kindly provided by E Brocchi, Istituto Zooprofilattico Sperimentale della Lombardia e dell'Emilia Romagna Reparto Biotecnologie, Italy) [37], IB11, FC6, AD10, BF8 and TRT1 [38] coupled to protein G sepharose. MAbs were subsequently screened by western blotting analysis and on FMDV vesicular lesions, non-infected tissue and on infected and mock-infected cells by immunofluorescence microscopy in combination with MAb 2C2 (anti-FMDV 3D) [10] and MAb 3C1 (anti-FMDV 3C) [11] kindly provided by E Brocchi.

### Immunofluorescence confocal microscopy

Samples collected at post-mortem were snap frozen in O.C.T. compound (Tissue-Tek) and stored at −80°C until processing. Acetone fixed frozen sections of acute tissue and tissue from 29 to 38 days post contact infection were labelled in duplicate with FMDV capsid MAbs, with consecutive sections labelled with MAbs 2C2, 3C1 [10], [11] and isotype control MAbs TRT1, TRT3 [38] and AV29, a MAb directed against a chicken antigen provided by F Davison, IAH. Additional sections were labelled with MAb D46 (specific for dark zone FDCs) [13], MAb CNA.42 (specific for light zone FDCs, kindly provided by G Delsol, Toulouse, CHU Purpan, Laboratoire d'anatomie et cytologie pathologiques, France) [13], MAb CC51 (anti-bovine CD21) [39], MAb 10D5 (anti-αvβ6, Chemicon) [12] and control MAb AV48, a MAb directed against a chicken antigen provided by F Davison, IAH. Anti-bovine CD32 MAbs CCG36 and CCG37 were kindly provided by C Howard, IAH. Goat anti-mouse Molecular Probes Alexa-Fluor-conjugated secondary MAbs (Invitrogen) were used and nuclei were stained with DAPI (Sigma). All data were collected sequentially using a Leica SP2 scanning laser confocal microscope.

### *In situ* hybridization

An optimised *In situ* hybridization method for the detection of FMDV was developed using digoxigenin-labelled RNA probes based on the protocol described by Prato Murphy *et al* [9] and optimised for frozen sections incorporating pre-hybridization blocking steps, tyramide signal amplification and alkaline phosphatase based visualization as described by Yang *et al* [40]. Consecutive frozen sections were simultaneously probed with; 500 nt 3D antisense and sense RNA probes specific for serotype O/UKG/34/2001 non-structural protein 3D coding sequence, IgG1 antisense RNA probe (686 nt) for the CH2, CH3 and hinge region of bovine IgG1 mRNA and a 615 nt swine vesicular disease virus probe specific for structural proteins 1C and 1D coding sequence [9].

## Supporting Information

Figure S1 Analysis of tissue 38 days post contact infection by LCM in combination with quantitative rRT-PCR. (A–C) Frozen sections stained with toluidine blue highlighting regions targeted during LCM. (A) Mandibular lymph node (MLN) and lateral retropharyngeal lymph node (RPLN) germinal centre and interfollicular regions targeted for microdissection. (B) Palatine tonsil (palatine t) germinal centre, interfollicular region, glandular epithelium and crypt epithelium targeted for microdissection. (C) Dorsal soft palate (DSP) and pharyngeal tonsil (pharyngeal t) germinal centres and epithelium targeted for microdissection. Three replicates of the different tissues regions, each containing six microdissected samples, were collected from each tissue and processed by quantitative rRT-PCR. Scale bars = 200 µm.
(6.74 MB TIF)

Figure S2 Dorsal soft palate samples analysed at 38 days post contact infection by LCM in combination with quantitative rRT-PCR to detect FMDV genome. FMDV genome was restricted to germinal centre samples (n = 4 animals, each bar represents six microdissected samples). No fluorescent signal above threshold was detected in epithelial samples by rRT-PCR after 50 cycles [33].
(0.68 MB TIF)

Figure S3 Pharyngeal tonsil samples analysed at 38 days post contact infection by LCM in combination with quantitative rRT-PCR to detect FMDV genome. FMDV genome was restricted to germinal centre samples (n = 4 animals, each bar represents six microdissected samples). No fluorescent signal above threshold was detected in epithelial samples by rRT-PCR after 50 cycles [33].
(0.67 MB TIF)

Figure S4 Palatine tonsil samples analysed at 38 days post contact infection by LCM in combination with quantitative rRT-PCR to detect FMDV genome. FMDV genome was restricted to germinal centre samples (n = 4 animals, each bar represents six microdissected samples). No fluorescent signal above threshold was detected in interfollicular, crypt epithelium or glandular epithelium samples by rRT-PCR after 50 cycles [33].
(1.14 MB TIF)

Figure S5 Lateral retropharyngeal lymph node samples analysed at 38 days post contact infection by LCM in combination with quantitative rRT-PCR to detect FMDV genome. FMDV genome was restricted to germinal centre samples (n = 4 animals, each bar represents six microdissected samples). No fluorescent signal above threshold was detected in interfollicular samples by rRT-PCR after 50 cycles [33].
(0.70 MB TIF)

Figure S6 Mandibular lymph node samples analysed at 38 days post contact infection by LCM in combination with quantitative rRT-PCR to detect FMDV genome. FMDV genome was restricted to germinal centre samples (n = 4 animals, each bar represents six microdissected samples). No fluorescent signal above threshold was detected in interfollicular samples by rRT-PCR after 50 cycles [33].
(0.70 MB TIF)

Figure S7 *In situ* hybridization detection protocol: comparison of tyramide signal amplification with conventional chromagenic detection. Detection protocols were compared and optimised on consecutive pharyngeal tonsil frozen sections using IgG1 RNA probes. (A) IgG1 antisense probe detected with tyramide signal amplification protocol showing deposits of blue-back chromagen in target cells with low background after developing for 2 minutes. (B) IgG1 antisense probe detected with conventional chromagen protocol [9] after developing for 2 minutes. No blue-black deposit could be seen. (C) IgG1 antisense probe detected with conventional chromagen protocol [9] after developing for 30 minutes. Deposits of blue-back chromagen can be seen in target cells but high background signal make the detection of rare mRNA difficult. (D) Background signal associated with IgG1 antisense probe and tyramide signal amplification after developing for 30 minutes. Scale bars, (A, C, D) = 500 µm, (B) = 200 µm.
(10.44 MB TIF)

Figure S8 3D antisense RNA probe validation on infected and mock-infected BHK-21 cells. (A) Positive signal following hybridization with 3D antisense RNA probe on BHK-21 cells fixed 5 hours after FMDV O/UKG/34/2001 infection at MOI 10. (B) Lack of specific signal on infected cells with SVD antisense probe. (C) Lack of specific signal on mock-infected cells following hybridization with 3D antisense probe. (D) Positive, cytoplasmic blue-black chromagen deposit on infected cells following hybridization with 3D antisense probe. (E) Faint blue-black chromagen deposit following hybridization with 3D sense probe 5 hours after FMDV infection at MOI 10. Scale bars, (A, B) = 500 µm, (C, D, E) = 25 µm.
(3.49 MB TIF)

Figure S9 *In situ* hybridization validation: 3D antisense RNA probe used on frozen sections 4 days post infection. Tissue samples were collected from animals 4 days post contact challenge. (A–B) Positive staining of coronary band epithelium following hybridization with 3D antisense RNA probe. (C–D) Lack of staining of coronary band epithelium following hybridization with SVD antisense and 3D sense RNA probes. No signal was detected in sections from non-infected control animals (data not shown). Scale bars, (A) = 200 µm, (B) = 50 µm, (C, D) = 500 µm.
(4.49 MB TIF)

Figure S10 Detection of FMDV capsid proteins in cell culture. SDS-PAGE analysis of virus infected (+) or mock-infected (−) BHK-21 cell lysates immunoprecipitated with MAb D9 (+ve control) [37], MAb IB11, BF8, AD10, FC6 and TRT1 (−ve control) [38]. MAbs IB11, BF8, AD10 and FC6 did not detect linearised FMDV by western blotting analysis (data not shown).
(0.47 MB TIF)

Figure S11 Tongue epithelium frozen sections from infected and non-infected animals labelled for immunofluorescence confocal microscopy. (A–F) Infected tongue epithelium frozen sections 4 days post contact challenge. (A) FMDV capsid proteins labelled green with MAb IB11. (B) FMDV non-structural protein 3A labelled red with MAb 2C2 [10]. (C) Co-localisation of FMDV capsid and 3A proteins. (D–F) No signal was detected on infected tongue epithelium with isotype control MAbs TRT1 (D), or TRT3 (E) [38]. (G–I) No signal was detected with MAbs IB11 (G), or 2C2 (H) on non-infected control tissue. Nuclei stained blue (DAPI), scale bars = 80 µm (MAbs FC6, AD10, BF8 and FMDV anti-3C MAb 3C1 showed a similar labelling pattern, data not shown) [11].
(10.11 MB TIF)

Figure S12 Infected and mock-infected BHK-21 cells labelled for immunofluorescence confocal microscopy. (A) FMDV capsid proteins labelled green with MAb IB11 on cells fixed 5 hours after FMDV O/UKG/34/2001 infection at MOI 10. (B) FMDV non-structural protein 3A labelled red with MAb 2C2 [10]. (C) Co-localisation of FMDV capsid and 3A proteins. (D) No signal was detected with MAbs IB11 or 2C2 on mock-infected cells. (E) No signal was detected on infected cells with isotype control MAbs TRT1 or TRT3 [38]. Nuclei stained blue (DAPI), scale bars, (A, B, C) = 5 µm, (D, E) = 10 µm (MAbs FC6, AD10, BF8 and FMDV anti-3C MAb 3C1 showed a similar labelling pattern, data not shown) [11].
(7.46 MB TIF)

Figure S13 Mandibular lymph node frozen sections labelled to detect FMDV proteins four days post contact challenge with O/UKG/34/2001. (A–C) A small number of cells were detected with MAbs to FMDV structural and non-structural proteins in the lymph node cortex during the acute stages of FMDV infection. (A) FMDV non-structural protein 3A labelled red with MAb 2C2 [10]. (B) FMDV capsid proteins labelled green with MAb IB11. (C) Co-localisation of FMDV capsid and 3A proteins. (D–F) Higher power image of lymph node cortex showing cytoplasmic FMDV capsid and 3A protein co-localisation during the acute stages of infection. No signal was detected with isotype control MAbs TRT1 or TRT3 [38] or with MAbs IB11 and 2C2 on non-infected control tissue (data not shown). Nuclei stained blue (DAPI), scale bars, (A, B, C) = 100 µm, (D, E, F) = 20 µm.
(2.22 MB TIF)

Figure S14 Detection of FMDV immune complexes in vitro. (A–C) Mouse fibroblast cells (3T3 cells) expressing bovine CD32 were paraformaldehyde fixed, washed and incubated with FMDV immune complexes prepared by incubating FMDV with heat inactivated bovine polyclonal immune serum. Cells were subsequently washed, fixed and labelled. (A) FMDV capsid protein labelled green with MAb IB11. (B) CD32 labelled red with MAb CCG37. (D–F) Cells prepared as above except FMDV was incubated with non-immune control serum. (D) No FMDV capsid proteins were detected. (G–I) Cells prepared as above with FMDV immune complexes and labelled. (G) No FMDV non-structural protein 3A was detected with MAb 2C2 [10], consistent with lack of replication and internalisation by fixed cells. (H) CD32 labelled red with MAb CCG36. Nuclei stained blue (DAPI), scale bars = 10 µm.
(10.08 MB TIF)

Figure S15 High power images of FMDV detected in mandibular lymph nodes 38 days post contact infection by *in situ* hybridization and immunohistochemical analysis. (A–C) Mandibular lymph node frozen sections analysed by *in situ* hybridization with (A) 3D antisense RNA probe, (B) SVD antisense RNA control probe and (C) 3D sense RNA control probe. No counterstain, scale bar = 50 µm. (D–F) Mandibular lymph node frozen sections labelled for immunofluorescence confocal microscopy. (D) MAb IB11 labelling FMDV capsid (green). (E) Isotype matched negative control MAb TRT1 [38]. (F) Lack of signal on non-infected control tissue labelled with IB11. Nuclei stained blue (DAPI), scale bars = 5 µm. Panel (A) and (D) highlight the similar diffuse punctate pattern using *in situ* hybridization to detect FMDV genome and MAb IB11 to detect FMDV capsid proteins.
(10.45 MB TIF)

Figure S16 The αvβ6 integrin is not expressed in germinal centres. (A–F) Palatine tonsil frozen sections. (A) Palatine tonsil crypt epithelium cells express the αvβ6 integrin labelled green with MAB 10D5 [12], green fluorescence in the adjacent germinal centre is due to autofluorescence associated with bovine germinal centres. No αvβ6 expression was seen in germinal centres. (B) CD21 expressing cells labelled with MAb CC51 [39]. (D–E) Consecutive frozen sections labelled with isotype control MAb TRT3 (D) [38] and AV29 (E). Nuclei stained blue (DAPI), scale bar = 100 µm.
(9.67 MB TIF)
