# Supplementary figures and images for: Foot-and-Mouth Disease Virus Persists in the Light Zone of Germinal Centres
Source: PLoS One. 2008 Oct 20;3(10):e3434. doi: 10.1371/journal.pone.0003434 (PMC2563691; doi:10.1371/journal.pone.0003434)

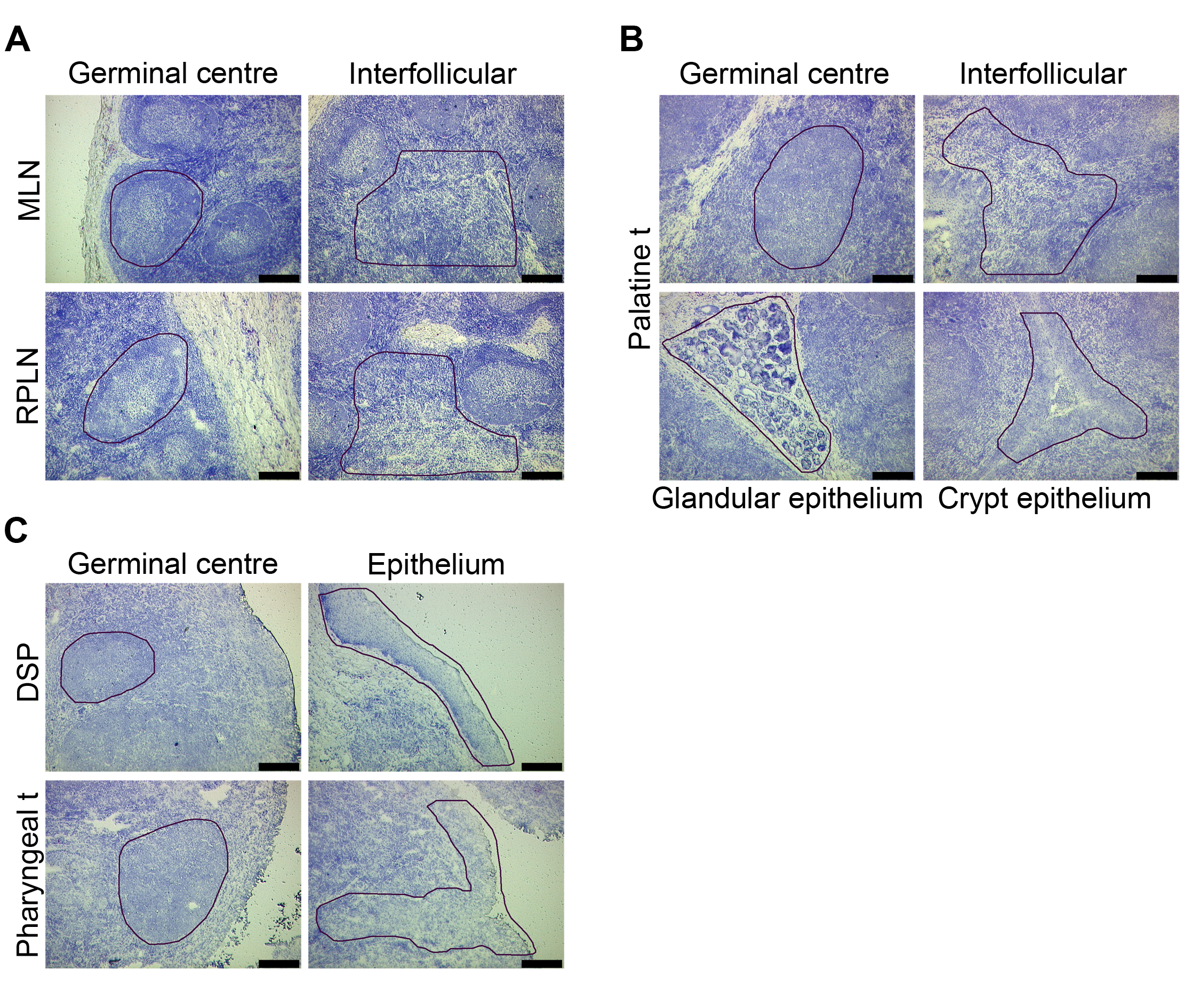

Supplement: Figure S1 — Analysis of tissue 38 days post contact infection by LCM in combination with quantitative rRT-PCR. (A–C) Frozen sections stained with toluidine blue highlighting regions targeted during LCM. (A) Mandibular lymph node (MLN) and lateral retropharyngeal lymph node (RPLN) germinal centre and interfollicular regions targeted for microdissection. (B) Palatine tonsil (palatine t) germinal centre, interfollicular region, glandular epithelium and crypt epithelium targeted for microdissection. (C) Dorsal soft palate (DSP) and pharyngeal tonsil (pharyngeal t) germinal centres and epithelium targeted for microdissection. Three replicates of the different tissues regions, each containing six microdissected samples, were collected from each tissue and processed by quantitative rRT-PCR. Scale bars = 200 µm. (6.74 MB TIF) [file pone.0003434.s001.tif]

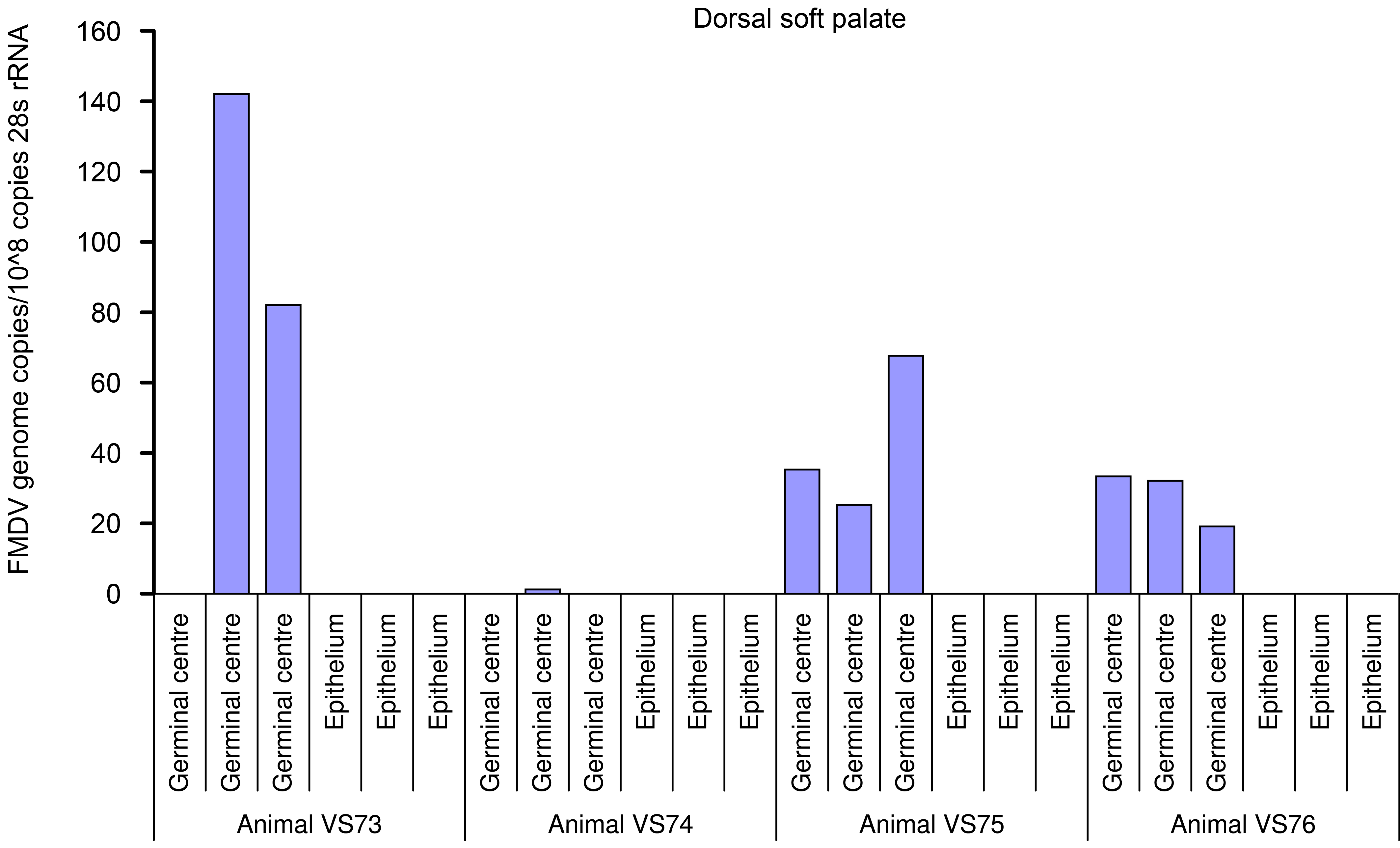

Supplement: Figure S2 — Dorsal soft palate samples analysed at 38 days post contact infection by LCM in combination with quantitative rRT-PCR to detect FMDV genome. FMDV genome was restricted to germinal centre samples (n = 4 animals, each bar represents six microdissected samples). No fluorescent signal above threshold was detected in epithelial samples by rRT-PCR after 50 cycles [33]. (0.68 MB TIF) [file pone.0003434.s002.tif]

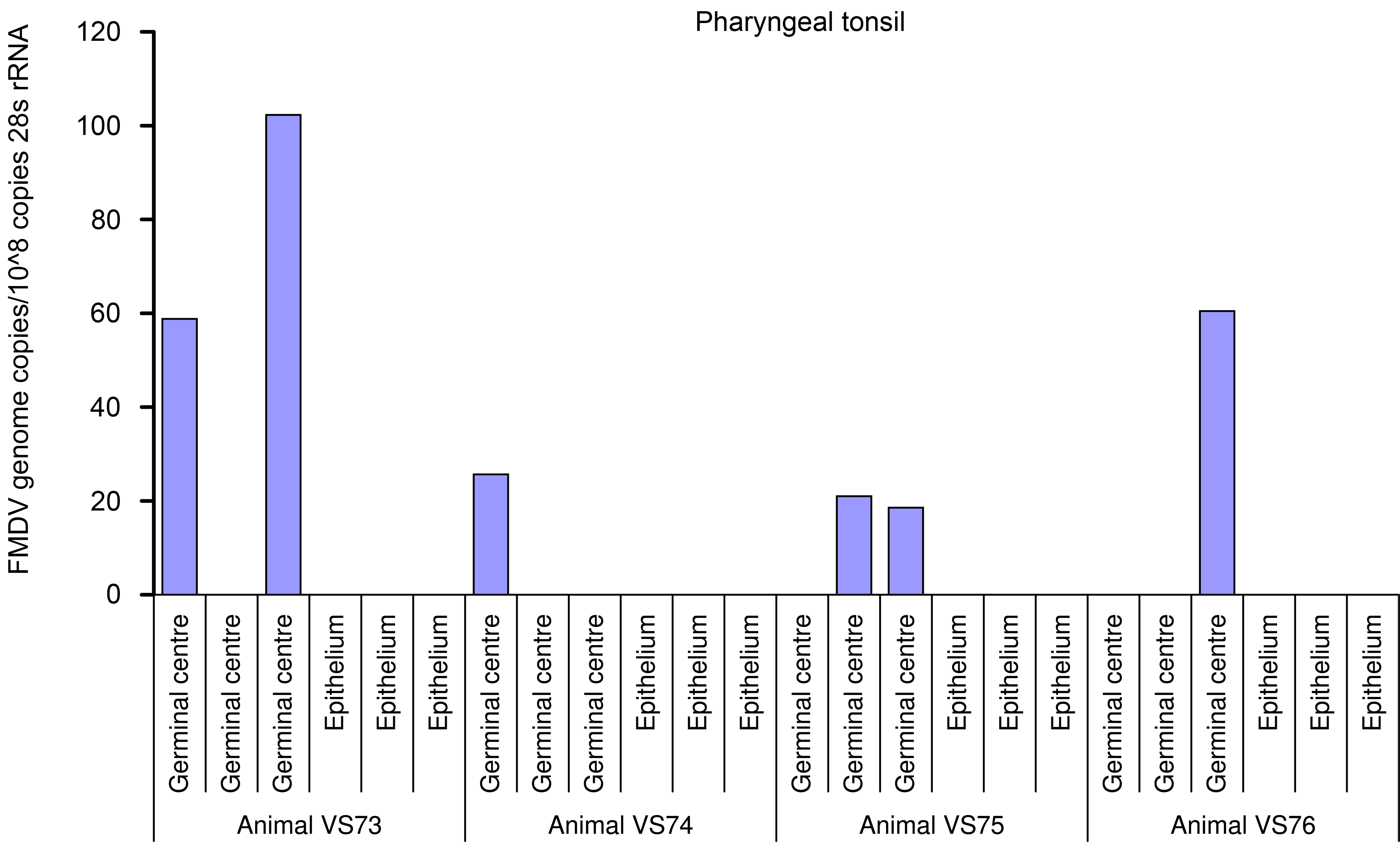

Supplement: Figure S3 — Pharyngeal tonsil samples analysed at 38 days post contact infection by LCM in combination with quantitative rRT-PCR to detect FMDV genome. FMDV genome was restricted to germinal centre samples (n = 4 animals, each bar represents six microdissected samples). No fluorescent signal above threshold was detected in epithelial samples by rRT-PCR after 50 cycles [33]. (0.67 MB TIF) [file pone.0003434.s003.tif]

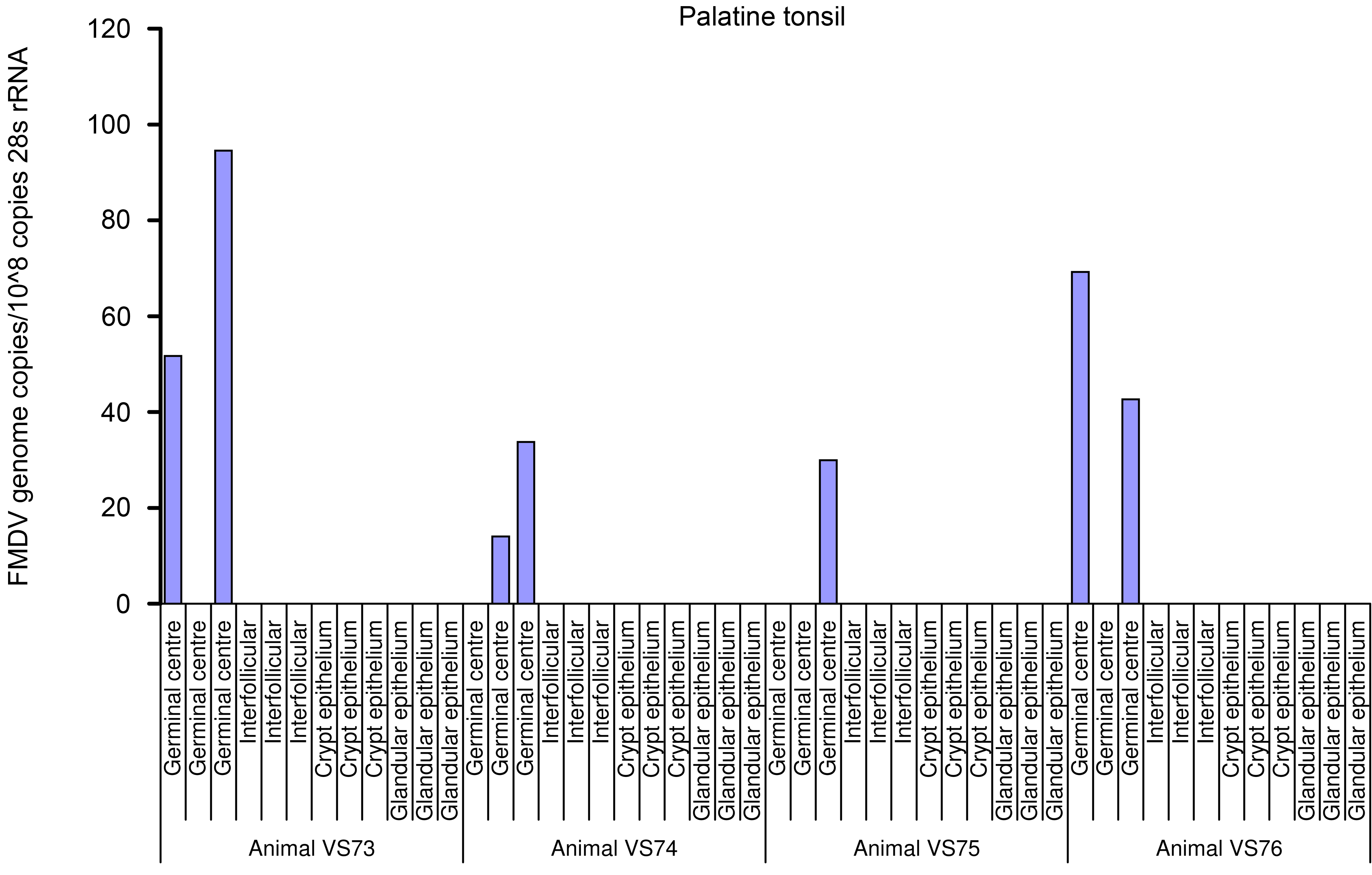

Supplement: Figure S4 — Palatine tonsil samples analysed at 38 days post contact infection by LCM in combination with quantitative rRT-PCR to detect FMDV genome. FMDV genome was restricted to germinal centre samples (n = 4 animals, each bar represents six microdissected samples). No fluorescent signal above threshold was detected in interfollicular, crypt epithelium or glandular epithelium samples by rRT-PCR after 50 cycles [33]. (1.14 MB TIF) [file pone.0003434.s004.tif]

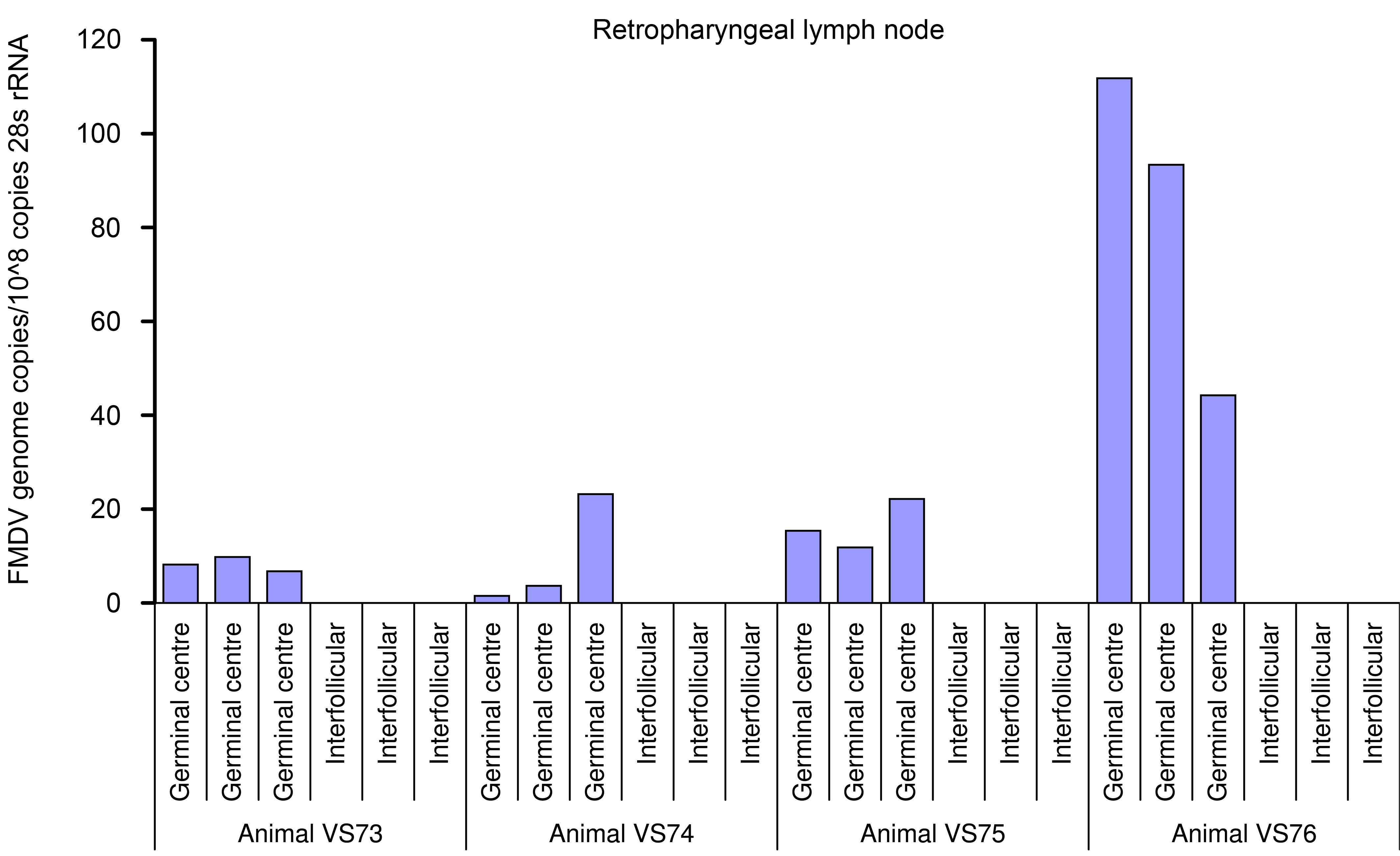

Supplement: Figure S5 — Lateral retropharyngeal lymph node samples analysed at 38 days post contact infection by LCM in combination with quantitative rRT-PCR to detect FMDV genome. FMDV genome was restricted to germinal centre samples (n = 4 animals, each bar represents six microdissected samples). No fluorescent signal above threshold was detected in interfollicular samples by rRT-PCR after 50 cycles [33]. (0.70 MB TIF) [file pone.0003434.s005.tif]

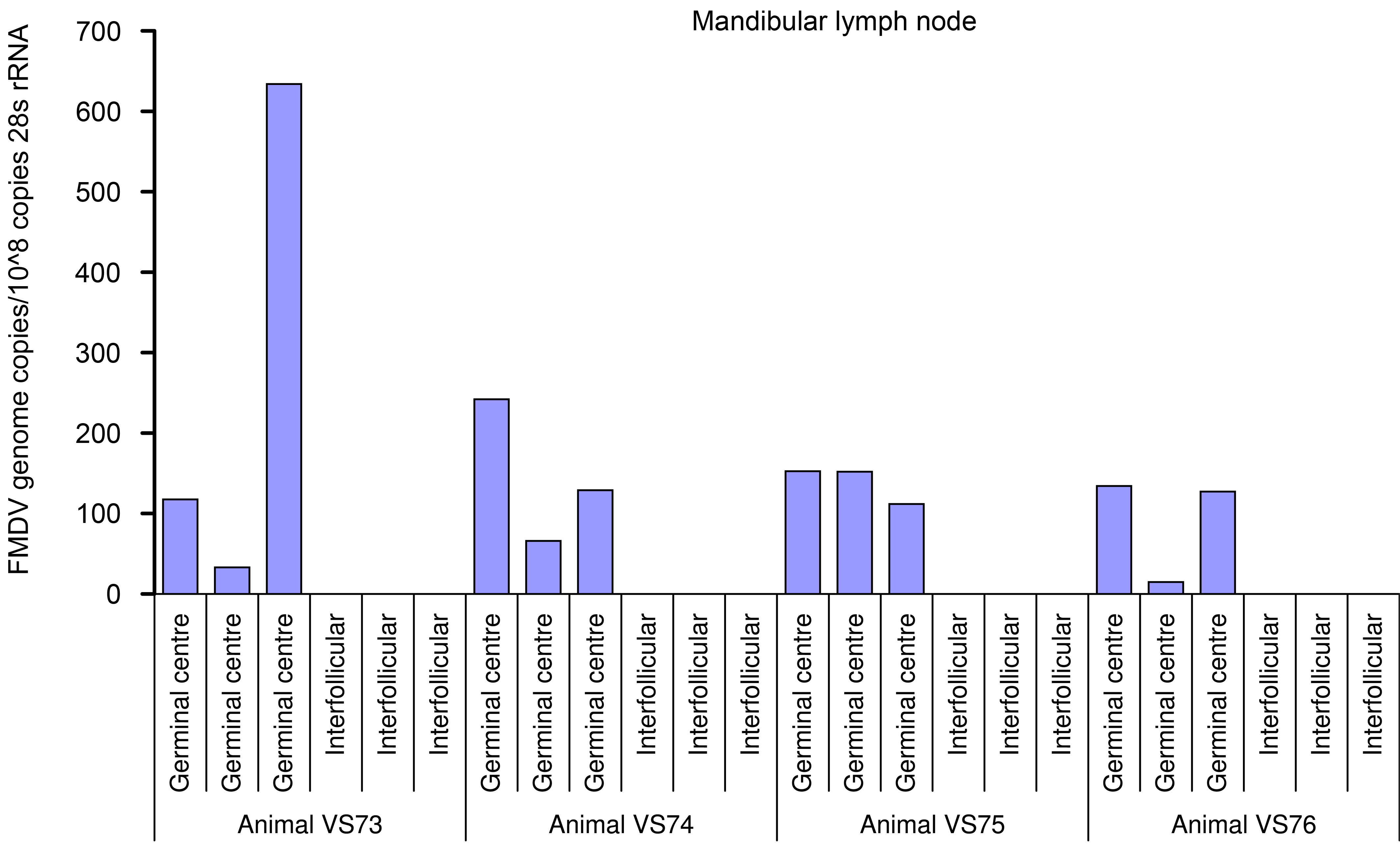

Supplement: Figure S6 — Mandibular lymph node samples analysed at 38 days post contact infection by LCM in combination with quantitative rRT-PCR to detect FMDV genome. FMDV genome was restricted to germinal centre samples (n = 4 animals, each bar represents six microdissected samples). No fluorescent signal above threshold was detected in interfollicular samples by rRT-PCR after 50 cycles [33]. (0.70 MB TIF) [file pone.0003434.s006.tif]

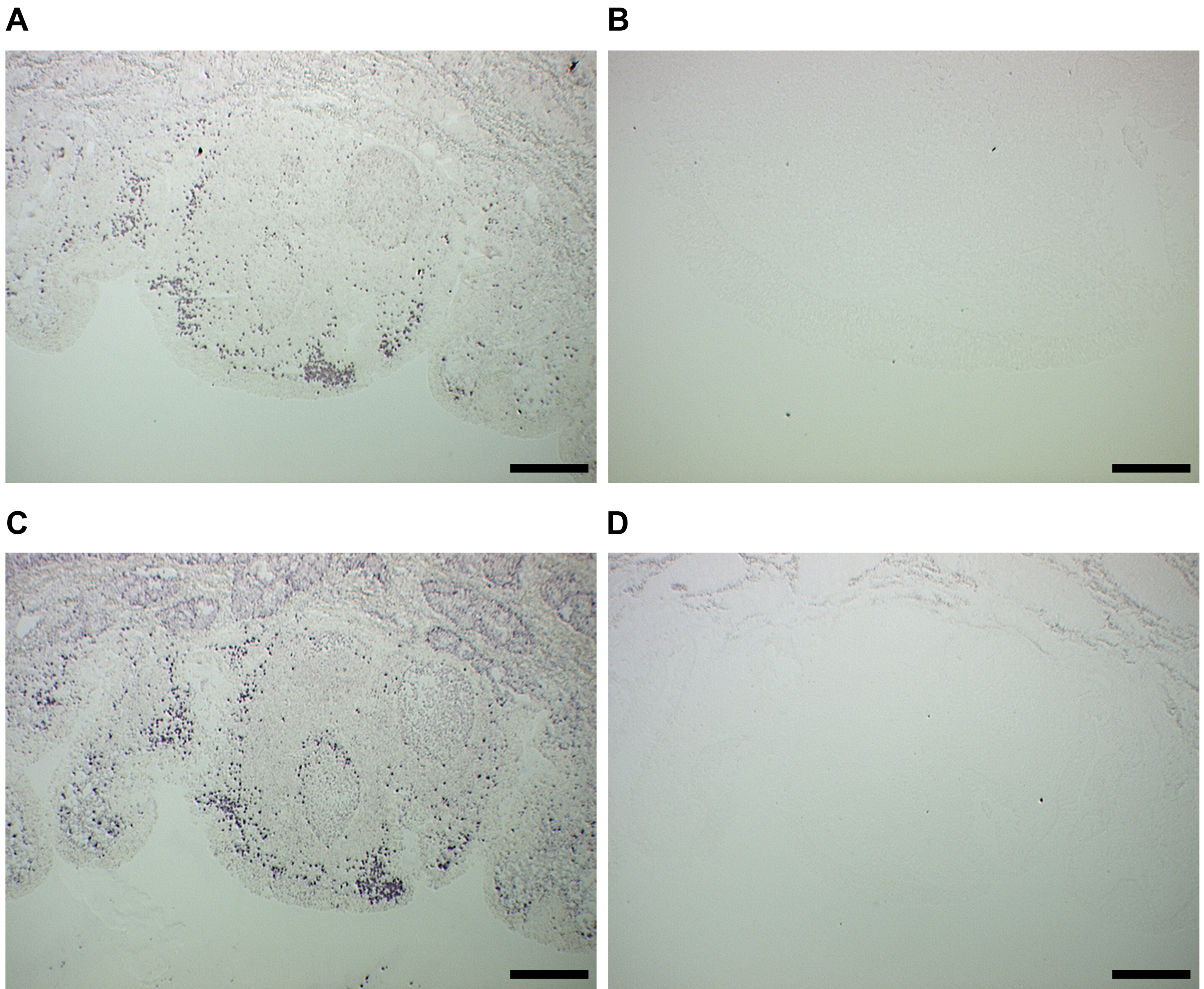

Supplement: Figure S7 — In situ hybridization detection protocol: comparison of tyramide signal amplification with conventional chromagenic detection. Detection protocols were compared and optimised on consecutive pharyngeal tonsil frozen sections using IgG1 RNA probes. (A) IgG1 antisense probe detected with tyramide signal amplification protocol showing deposits of blue-back chromagen in target cells with low background after developing for 2 minutes. (B) IgG1 antisense probe detected with conventional chromagen protocol [9] after developing for 2 minutes. No blue-black deposit could be seen. (C) IgG1 antisense probe detected with conventional chromagen protocol [9] after developing for 30 minutes. Deposits of blue-back chromagen can be seen in target cells but high background signal make the detection of rare mRNA difficult. (D) Background signal associated with IgG1 antisense probe and tyramide signal amplification after developing for 30 minutes. Scale bars, (A, C, D) = 500 µm, (B) = 200 µm. (10.44 MB TIF) [file pone.0003434.s007.tif]

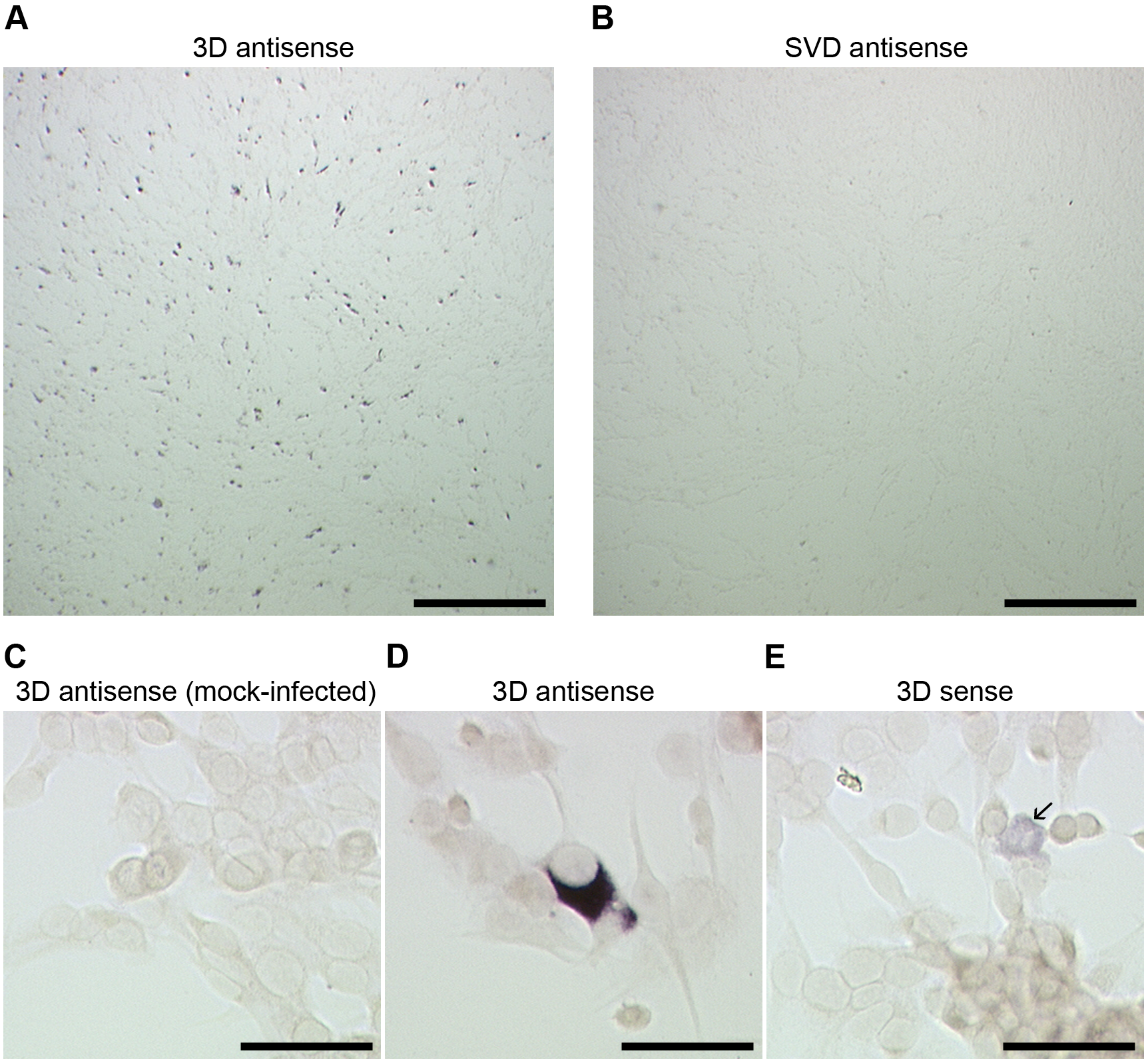

Supplement: Figure S8 — 3D antisense RNA probe validation on infected and mock-infected BHK-21 cells. (A) Positive signal following hybridization with 3D antisense RNA probe on BHK-21 cells fixed 5 hours after FMDV O/UKG/34/2001 infection at MOI 10. (B) Lack of specific signal on infected cells with SVD antisense probe. (C) Lack of specific signal on mock-infected cells following hybridization with 3D antisense probe. (D) Positive, cytoplasmic blue-black chromagen deposit on infected cells following hybridization with 3D antisense probe. (E) Faint blue-black chromagen deposit following hybridization with 3D sense probe 5 hours after FMDV infection at MOI 10. Scale bars, (A, B) = 500 µm, (C, D, E) = 25 µm. (3.49 MB TIF) [file pone.0003434.s008.tif]

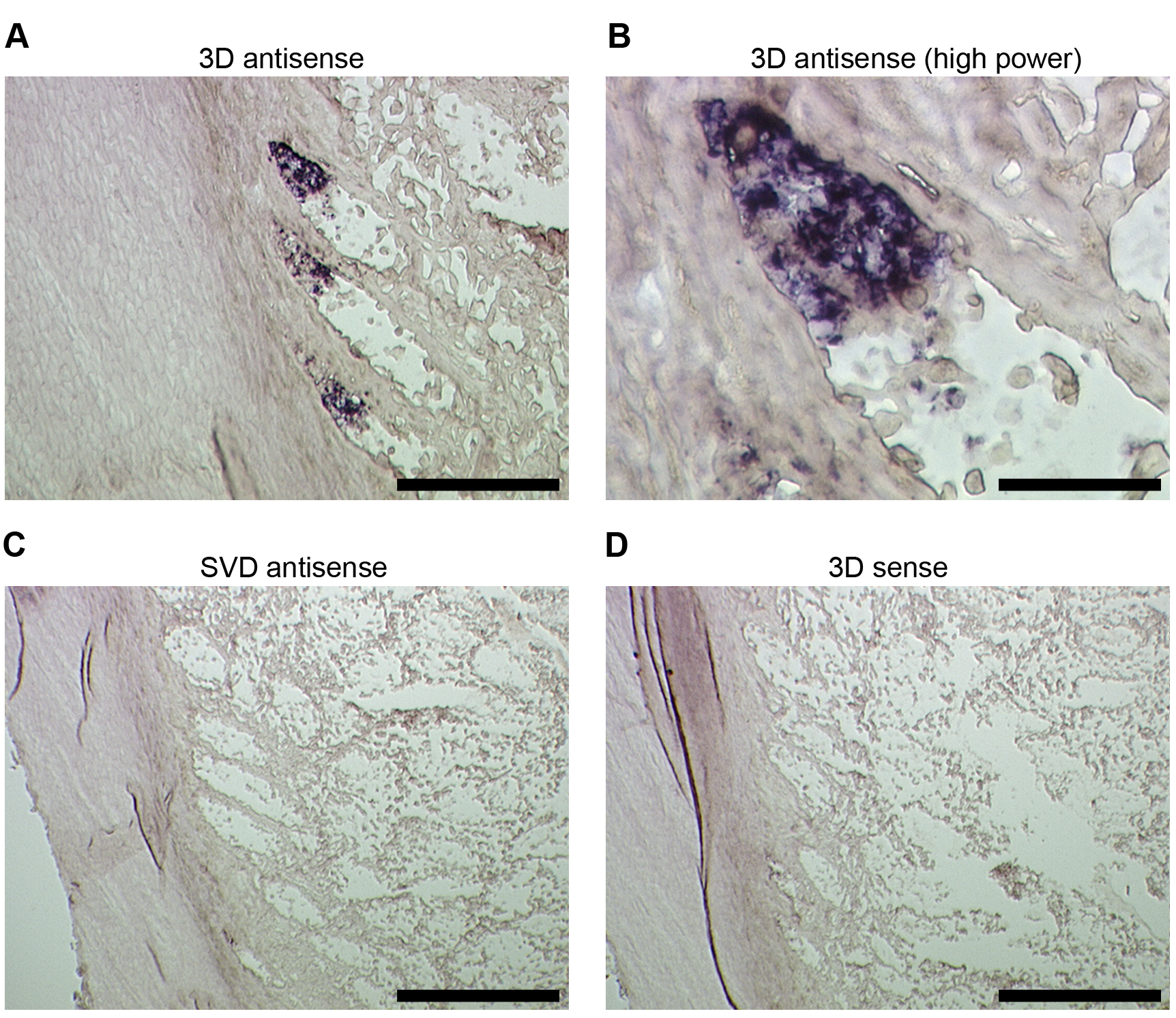

Supplement: Figure S9 — In situ hybridization validation: 3D antisense RNA probe used on frozen sections 4 days post infection. Tissue samples were collected from animals 4 days post contact challenge. (A–B) Positive staining of coronary band epithelium following hybridization with 3D antisense RNA probe. (C–D) Lack of staining of coronary band epithelium following hybridization with SVD antisense and 3D sense RNA probes. No signal was detected in sections from non-infected control animals (data not shown). Scale bars, (A) = 200 µm, (B) = 50 µm, (C, D) = 500 µm. (4.49 MB TIF) [file pone.0003434.s009.tif]

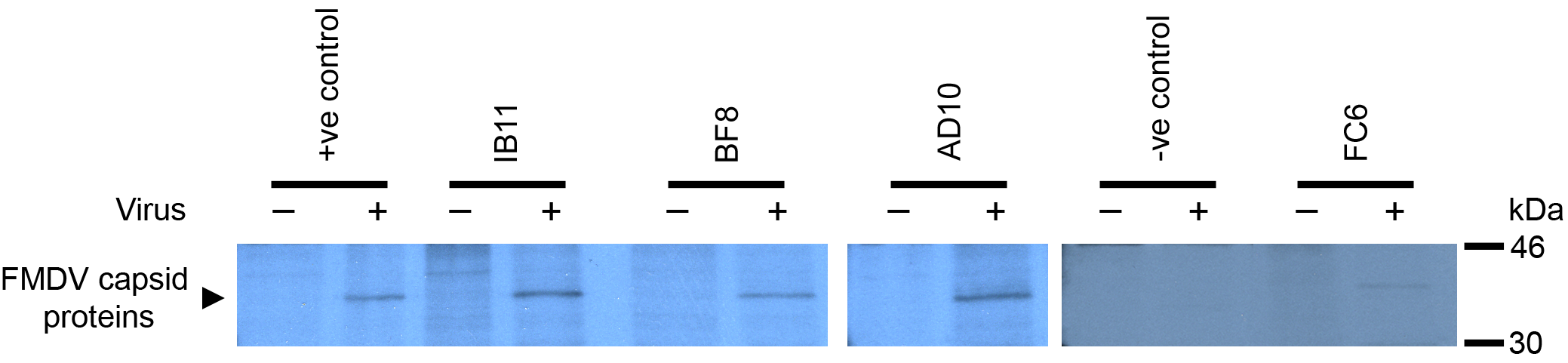

Supplement: Figure S10 — Detection of FMDV capsid proteins in cell culture. SDS-PAGE analysis of virus infected (+) or mock-infected (−) BHK-21 cell lysates immunoprecipitated with MAb D9 (+ve control) [37], MAb IB11, BF8, AD10, FC6 and TRT1 (−ve control) [38]. MAbs IB11, BF8, AD10 and FC6 did not detect linearised FMDV by western blotting analysis (data not shown). (0.47 MB TIF) [file pone.0003434.s010.tif]

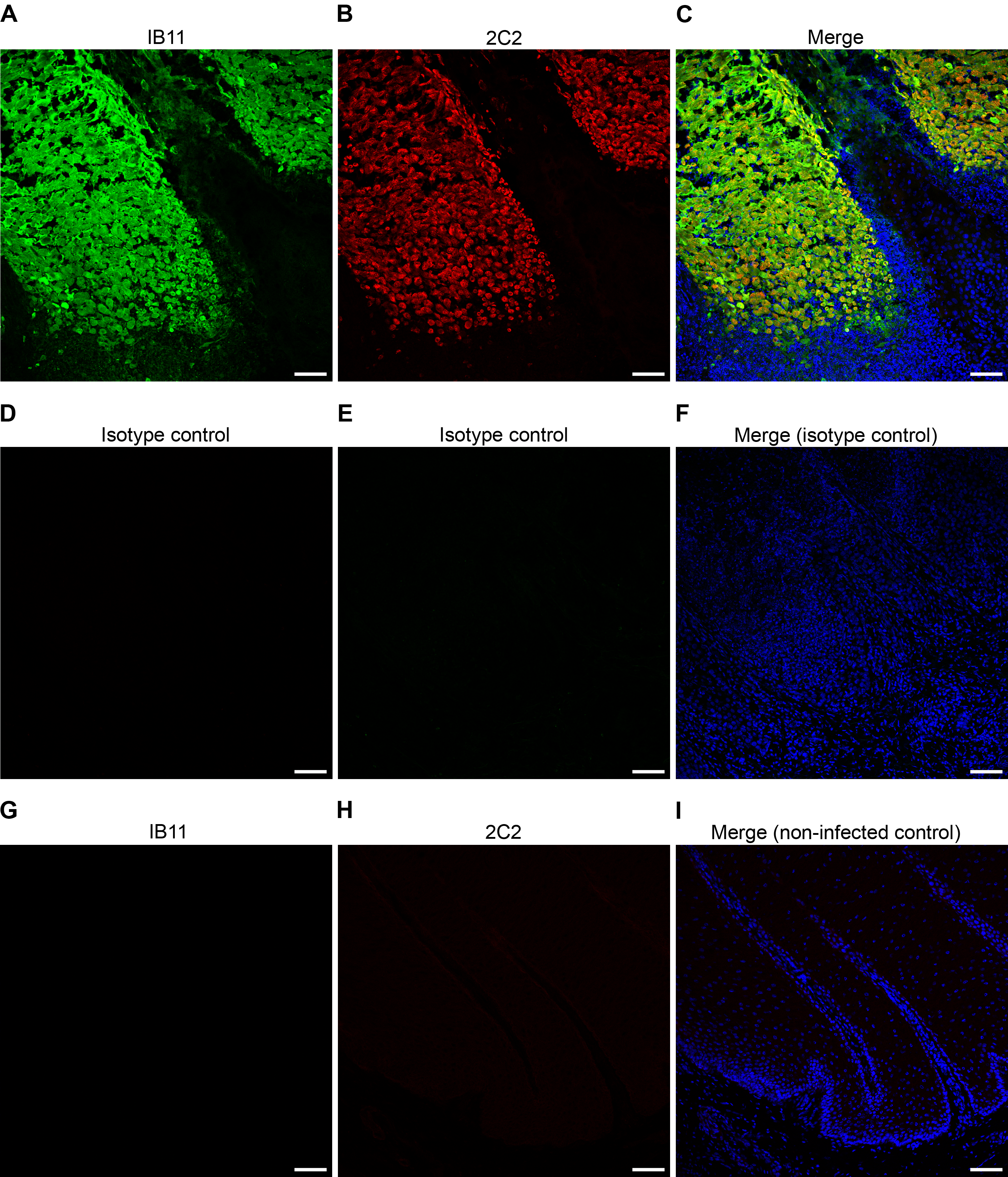

Supplement: Figure S11 — Tongue epithelium frozen sections from infected and non-infected animals labelled for immunofluorescence confocal microscopy. (A–F) Infected tongue epithelium frozen sections 4 days post contact challenge. (A) FMDV capsid proteins labelled green with MAb IB11. (B) FMDV non-structural protein 3A labelled red with MAb 2C2 [10]. (C) Co-localisation of FMDV capsid and 3A proteins. (D–F) No signal was detected on infected tongue epithelium with isotype control MAbs TRT1 (D), or TRT3 (E) [38]. (G–I) No signal was detected with MAbs IB11 (G), or 2C2 (H) on non-infected control tissue. Nuclei stained blue (DAPI), scale bars = 80 µm (MAbs FC6, AD10, BF8 and FMDV anti-3C MAb 3C1 showed a similar labelling pattern, data not shown) [11]. (10.11 MB TIF) [file pone.0003434.s011.tif]

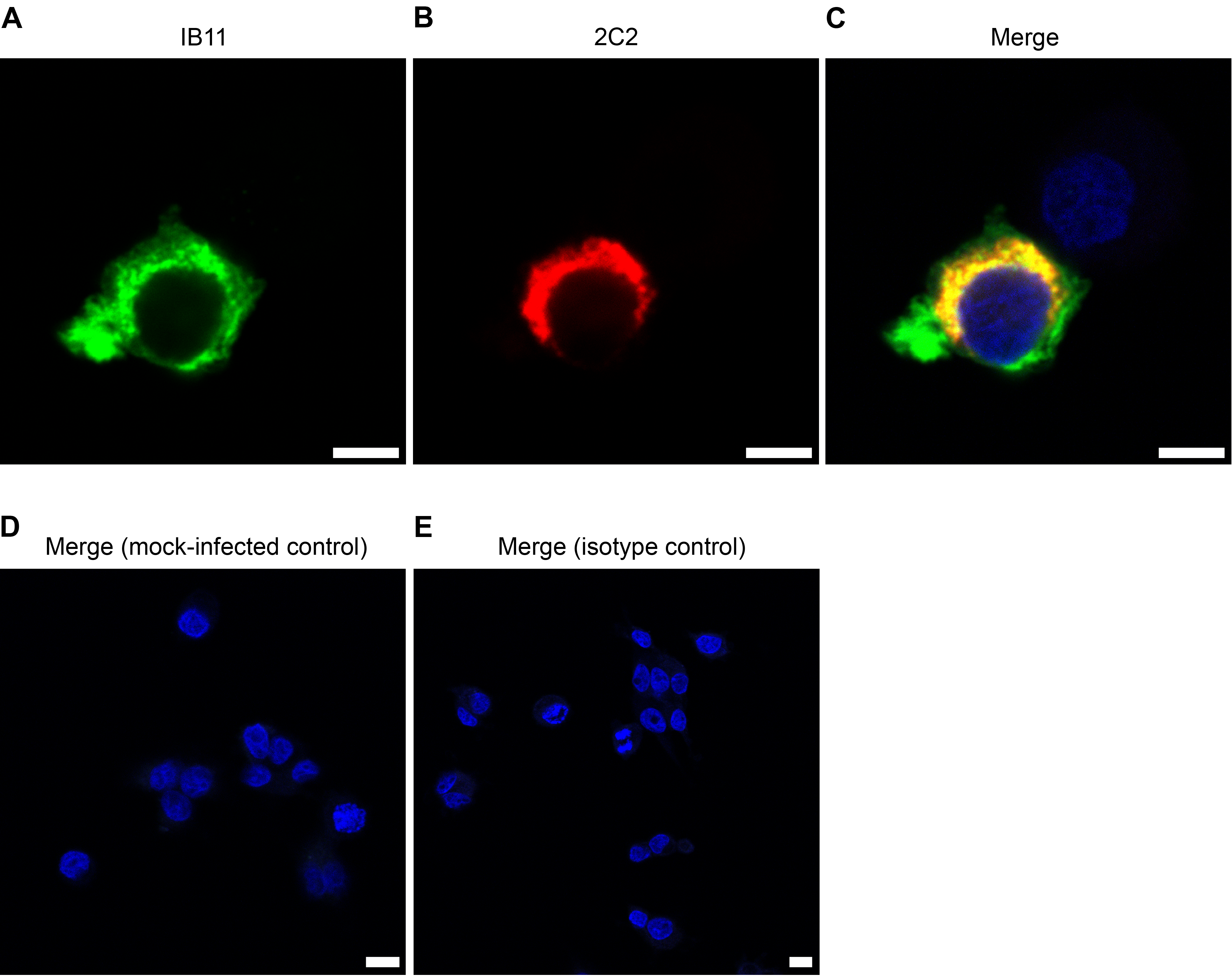

Supplement: Figure S12 — Infected and mock-infected BHK-21 cells labelled for immunofluorescence confocal microscopy. (A) FMDV capsid proteins labelled green with MAb IB11 on cells fixed 5 hours after FMDV O/UKG/34/2001 infection at MOI 10. (B) FMDV non-structural protein 3A labelled red with MAb 2C2 [10]. (C) Co-localisation of FMDV capsid and 3A proteins. (D) No signal was detected with MAbs IB11 or 2C2 on mock-infected cells. (E) No signal was detected on infected cells with isotype control MAbs TRT1 or TRT3 [38]. Nuclei stained blue (DAPI), scale bars, (A, B, C) = 5 µm, (D, E) = 10 µm (MAbs FC6, AD10, BF8 and FMDV anti-3C MAb 3C1 showed a similar labelling pattern, data not shown) [11]. (7.46 MB TIF) [file pone.0003434.s012.tif]

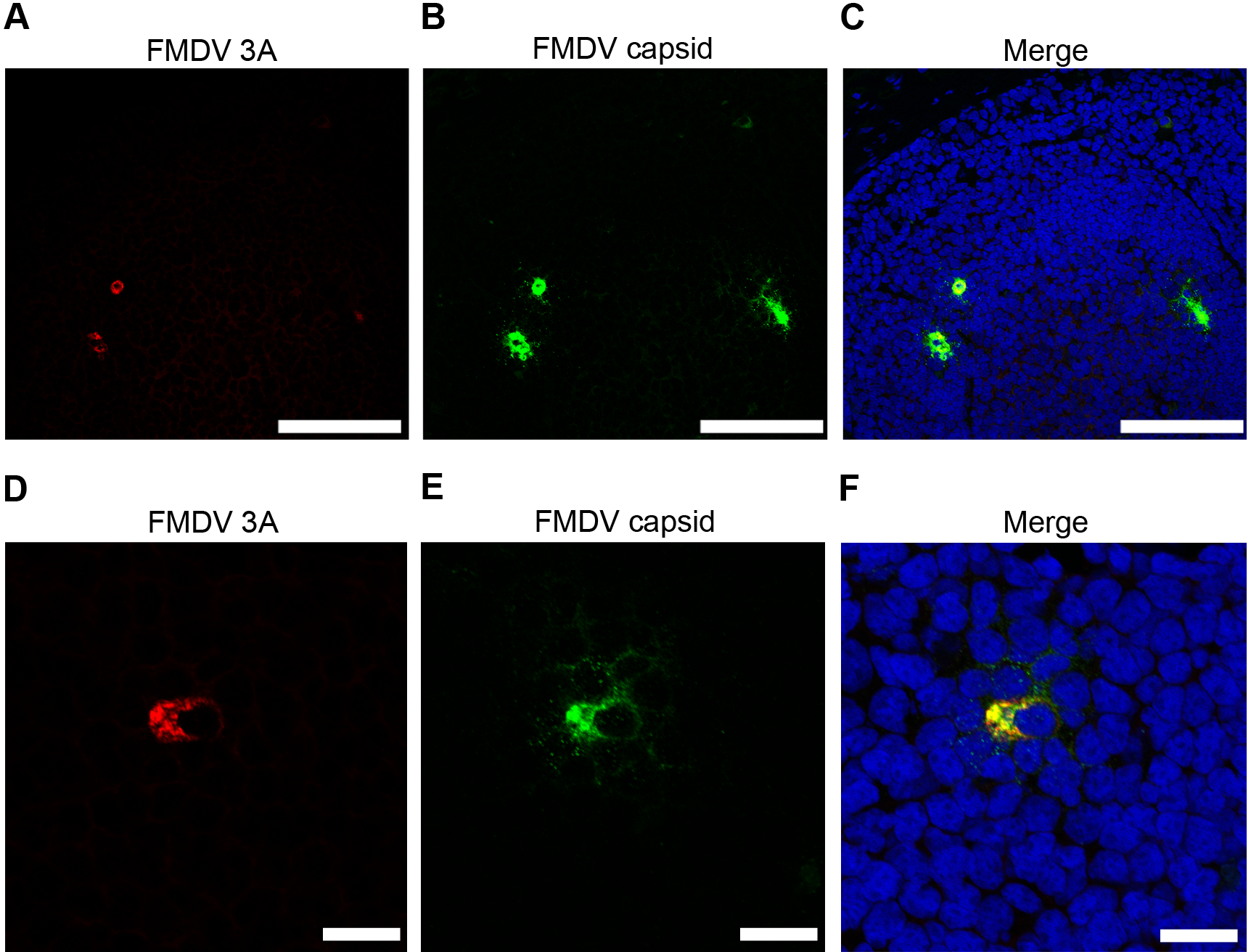

Supplement: Figure S13 — Mandibular lymph node frozen sections labelled to detect FMDV proteins four days post contact challenge with O/UKG/34/2001. (A–C) A small number of cells were detected with MAbs to FMDV structural and non-structural proteins in the lymph node cortex during the acute stages of FMDV infection. (A) FMDV non-structural protein 3A labelled red with MAb 2C2 [10]. (B) FMDV capsid proteins labelled green with MAb IB11. (C) Co-localisation of FMDV capsid and 3A proteins. (D–F) Higher power image of lymph node cortex showing cytoplasmic FMDV capsid and 3A protein co-localisation during the acute stages of infection. No signal was detected with isotype control MAbs TRT1 or TRT3 [38] or with MAbs IB11 and 2C2 on non-infected control tissue (data not shown). Nuclei stained blue (DAPI), scale bars, (A, B, C) = 100 µm, (D, E, F) = 20 µm. (2.22 MB TIF) [file pone.0003434.s013.tif]

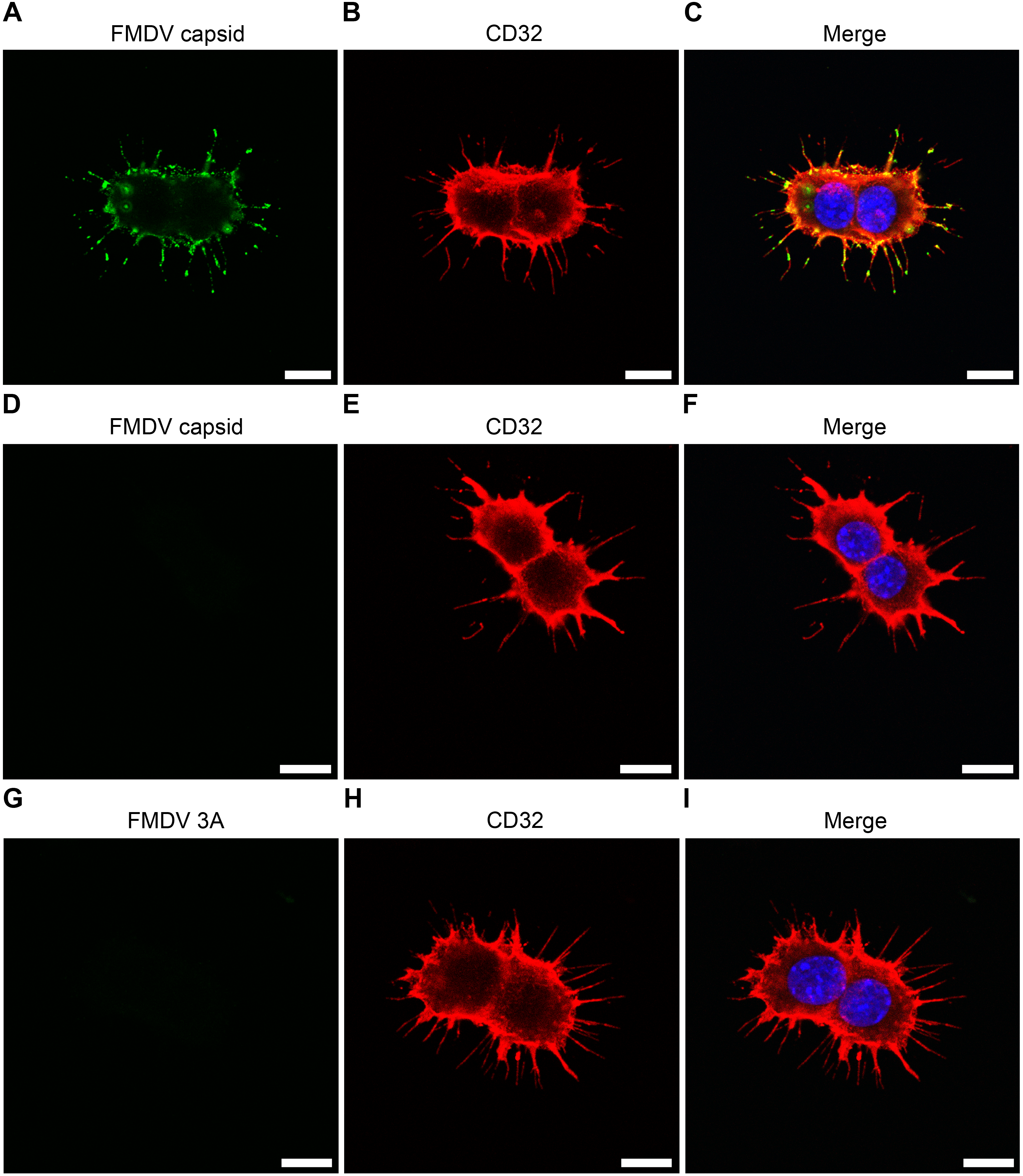

Supplement: Figure S14 — Detection of FMDV immune complexes in vitro. (A–C) Mouse fibroblast cells (3T3 cells) expressing bovine CD32 were paraformaldehyde fixed, washed and incubated with FMDV immune complexes prepared by incubating FMDV with heat inactivated bovine polyclonal immune serum. Cells were subsequently washed, fixed and labelled. (A) FMDV capsid protein labelled green with MAb IB11. (B) CD32 labelled red with MAb CCG37. (D–F) Cells prepared as above except FMDV was incubated with non-immune control serum. (D) No FMDV capsid proteins were detected. (G–I) Cells prepared as above with FMDV immune complexes and labelled. (G) No FMDV non-structural protein 3A was detected with MAb 2C2 [10], consistent with lack of replication and internalisation by fixed cells. (H) CD32 labelled red with MAb CCG36. Nuclei stained blue (DAPI), scale bars = 10 µm. (10.08 MB TIF) [file pone.0003434.s014.tif]

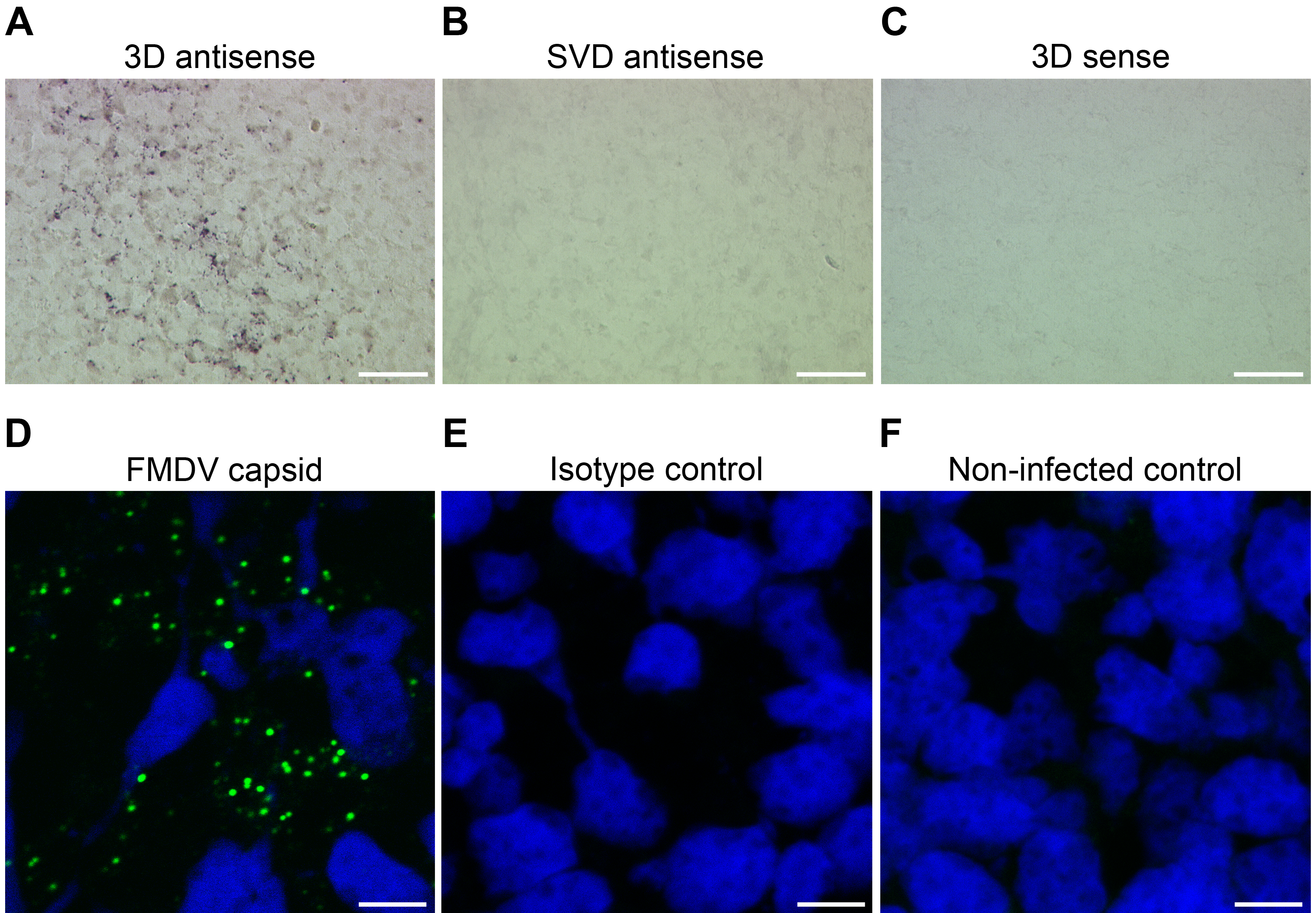

Supplement: Figure S15 — High power images of FMDV detected in mandibular lymph nodes 38 days post contact infection by in situ hybridization and immunohistochemical analysis. (A–C) Mandibular lymph node frozen sections analysed by in situ hybridization with (A) 3D antisense RNA probe, (B) SVD antisense RNA control probe and (C) 3D sense RNA control probe. No counterstain, scale bar = 50 µm. (D–F) Mandibular lymph node frozen sections labelled for immunofluorescence confocal microscopy. (D) MAb IB11 labelling FMDV capsid (green). (E) Isotype matched negative control MAb TRT1 [38]. (F) Lack of signal on non-infected control tissue labelled with IB11. Nuclei stained blue (DAPI), scale bars = 5 µm. Panel (A) and (D) highlight the similar diffuse punctate pattern using in situ hybridization to detect FMDV genome and MAb IB11 to detect FMDV capsid proteins. (10.45 MB TIF) [file pone.0003434.s015.tif]

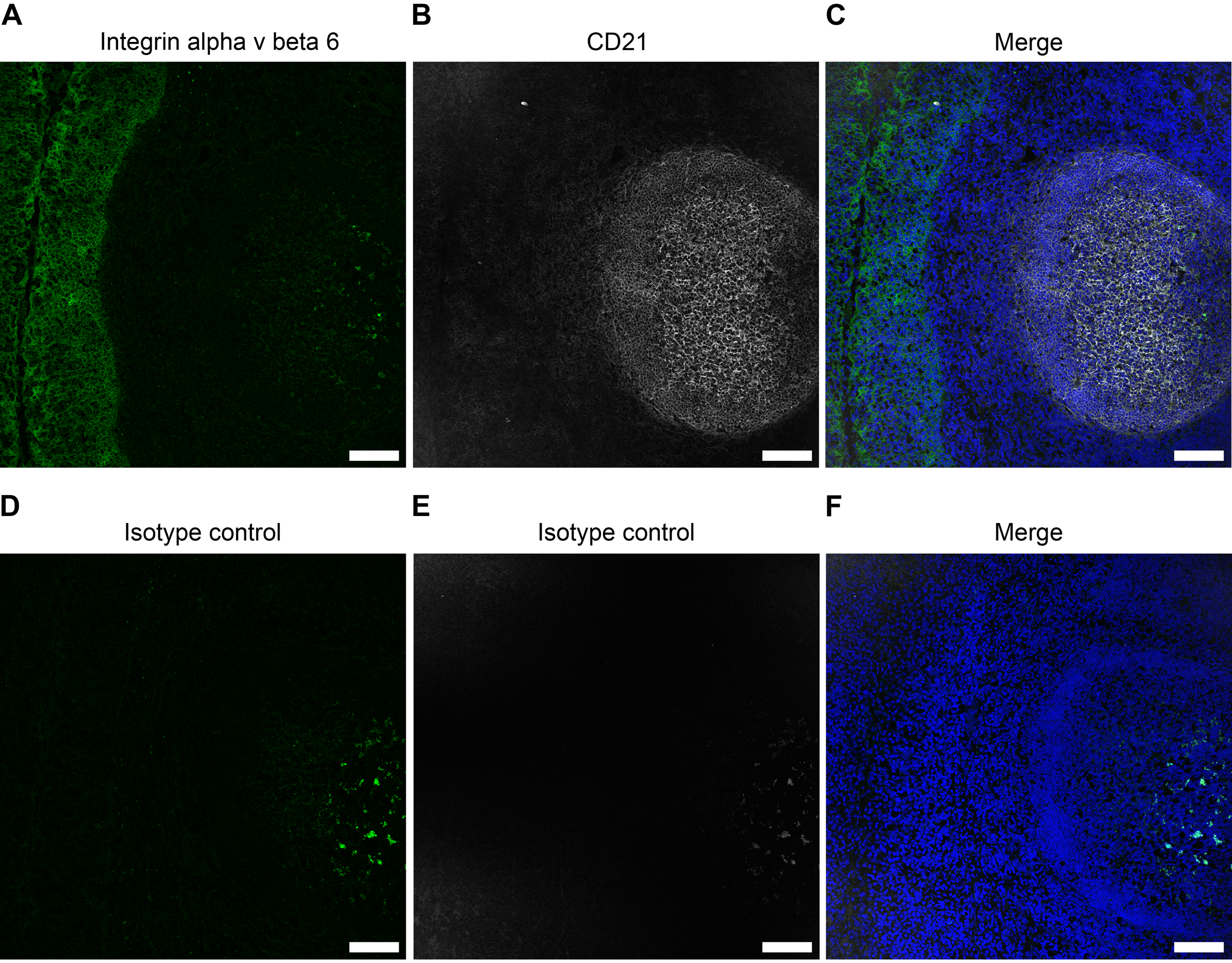

Supplement: Figure S16 — The αvβ6 integrin is not expressed in germinal centres. (A–F) Palatine tonsil frozen sections. (A) Palatine tonsil crypt epithelium cells express the αvβ6 integrin labelled green with MAB 10D5 [12], green fluorescence in the adjacent germinal centre is due to autofluorescence associated with bovine germinal centres. No αvβ6 expression was seen in germinal centres. (B) CD21 expressing cells labelled with MAb CC51 [39]. (D–E) Consecutive frozen sections labelled with isotype control MAb TRT3 (D) [38] and AV29 (E). Nuclei stained blue (DAPI), scale bar = 100 µm. (9.67 MB TIF) [file pone.0003434.s016.tif]
